# Modulation of the Self‐Assembly of π‐Amphiphiles in Water from Enthalpy‐ to Entropy‐Driven by Enwrapping Substituents

**DOI:** 10.1002/chem.202000995

**Published:** 2020-06-17

**Authors:** Pradeep P. N. Syamala, Frank Würthner

**Affiliations:** ^1^ Institut für Organische Chemie Universität Würzburg Am Hubland 97074 Würzburg Germany; ^2^ Center for Nanosystems Chemistry (CNC), & Bavarian Polymer Institute (BPI) Universität Würzburg Theodor-Boveri-Weg 97074 Würzburg Germany

**Keywords:** amphiphilic dyes, self-assembly, thermodynamics, OEG chains, π-conjugated systems

## Abstract

Depending on the connectivity of solubilizing oligoethylene glycol (OEG) side chains to the π‐cores of amphiphilic naphthalene and perylene bisimide dyes, self‐assembly in water occurs either upon heating or cooling. Herein, we show that this effect originates from differences in the enwrapping capability of the π‐cores by the OEG chains. Rylene bisimides bearing phenyl substituents with three OEG chains attached directly to the hydrophobic π‐cores are strongly sequestered by the OEG chains. These molecules self‐assemble at elevated temperatures in an entropy‐driven process according to temperature‐ and concentration‐dependent UV/Vis spectroscopy and calorimetric dilution studies. In contrast, for rylene bisimides in which phenyl substituents with three OEG chains are attached via a methylene spacer, leading to much weaker sequestration, self‐assembly originates upon cooling in an enthalpy‐driven process. Our explanation for this controversial behavior is that the aggregation in the latter case is dictated by the release of “high energy water” from the hydrophobic π‐surfaces as well as dispersion interactions between the π‐scaffolds which drive the self‐assembly in an enthalpically driven process. In contrast, for the former case we suggest that in addition to the conventional explanation of a dehydration of hydrogen‐bonded water molecules from OEG units it is in particular the increase in conformational entropy of back‐folded OEG side chains upon aggregation that provides the pronounced gain in entropy that drives the aggregation process. Thus, our studies revealed that a subtle change in the attachment of solubilizing substituents can switch the thermodynamic signature for the self‐assembly of amphiphilic dyes in water from enthalpy‐ to entropy‐driven.

## Graphical abstract

**Connectivity matters**: A de novo design strategy is developed to generate supramolecular polymers upon heating (entropically driven) or cooling (enthalpically driven) based on the connectivity of amphiphilic wedges to the hydrophobic π‐core. Structural characterizations point towards the unique orientation of side chains in guiding this thermodynamic bias of self‐assembly in water (see figure).

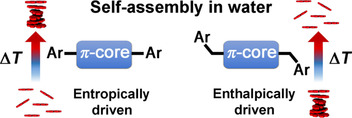

## Introduction

Self‐assembly has emerged as an efficient method for the development of functional materials possessing enticing properties, including self‐healing and stimuli‐responsiveness among many others.^1^ Such emergent functions of larger entities are encoded in the monomer design, which forecasts the dominant non‐covalent forces that take part in the formation of targeted architectures.^2^ In nature, these interactions are often not primarily governed by the specific non‐covalent bonds between the self‐assembling molecules but by the solvation properties of water, which plays a prominent role in the generation of these dynamic structures.^3^

Emulating nature's strategy for artificial supramolecular aggregates has proved to be challenging since our understanding of the role of water in these systems is still at its infancy.^4^ Nevertheless, a wide variety of nano‐scale structures in aqueous media has been generated, for example, for peptide amphiphiles^2^, ^5^ and π‐amphiphiles consisting of diverse hydrophobic cores including hexabenzocoronenes,^6^ naphthalene^7^ and perylene bisimides,3b, 8 benzene tri‐carboxylic acid,^9^ oligo‐phenylenes,^10^ phenylene ethynylenes,^11^ and phenylene vinylenes,^12^ etc.

**Figure 1:**
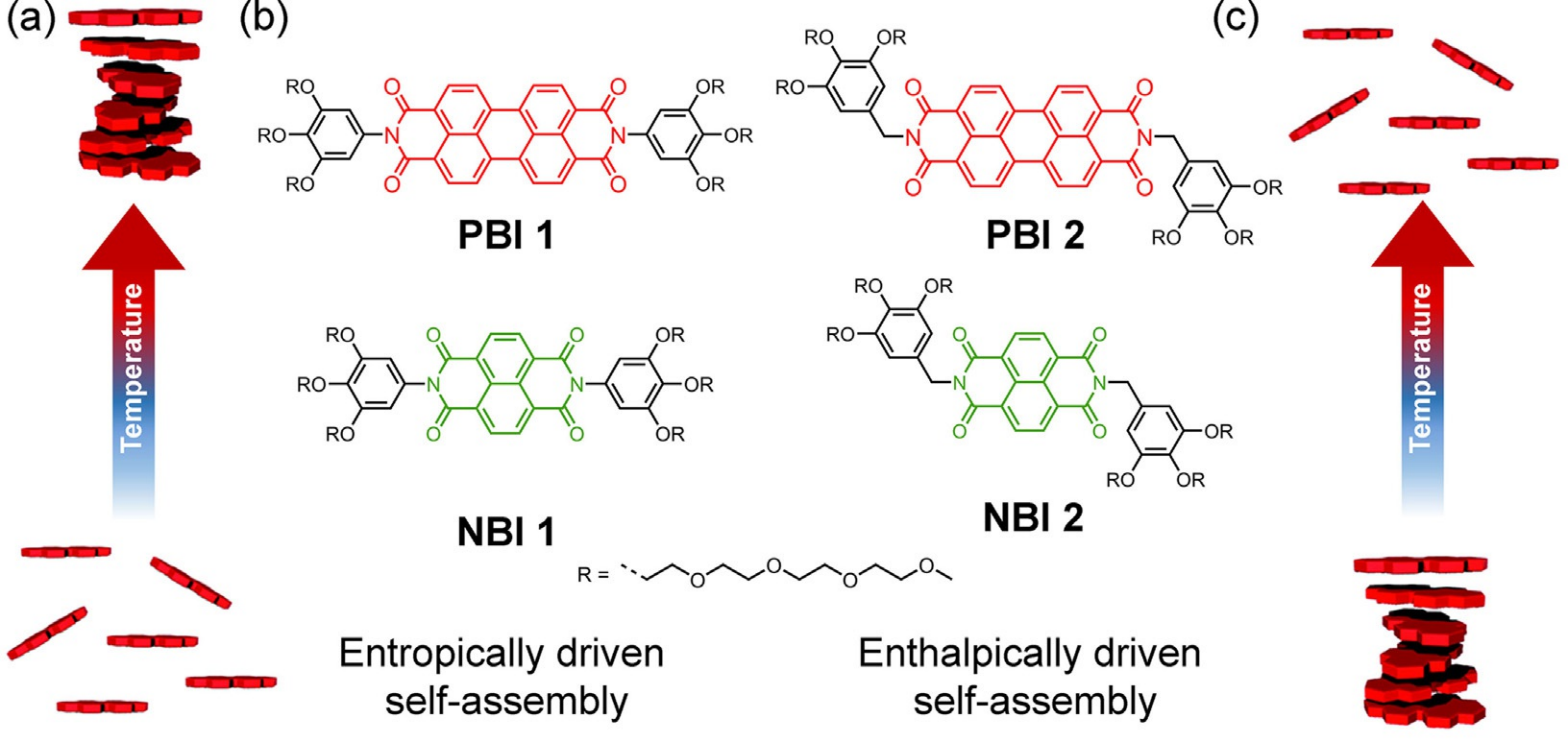
Schematic representation of entropically driven (a) and enthalpically driven (c) self‐assembly. (b) Chemical structures of **PBI 1**–**2** and **NBI 1**–**2**.

While the distinct mechanisms mediating the self‐assembly of aforementioned structures at room temperature in water has been explored in detail, providing insights into isodesmic, cooperative and anti‐cooperative models, understanding of thermodynamic factors which drive the self‐assembly, *viz*. enthalpy or entropy, is yet to transpire.^13^ This gap in the knowledge is probably related to the fact that the majority of the supramolecular aggregations are performed in organic media under the control of enthalpic factors (termed “ordinary temperature response”) assisted by non‐covalent bond formation and reduction in degrees of freedom associated with self‐assembly (Figure 1 c).^14^ However, in aqueous environments as given in nature, there are many examples for entropically driven self‐assembly processes (Figure 1 a) where the aggregation is favored at elevated temperatures (termed “inverse temperature response”) including tobacco mosaic virus,^15^ β‐amyloids,^16^ collagen fibrils,^17^ etc. In contrast, among synthetic supramolecular systems such cases are still rare.^14^, ^18^ Nevertheless, the design principles which underpin these bifurcated thermal responses can have wider implications ranging from the development of thermoresponsive materials to drug screening. For example, enthalpically driven drug‐protein interactions are known to evade undesirable physicochemical properties^19^ and ligands that bind with an entropic advantage can adopt multiple binding modes and thus circumvent the development of resistance.^20^ This, in turn, calls for a detailed understanding of the enthalpic and entropic factors that govern self‐assembly and rational design strategies which can encode this information into monomeric building blocks.

We have previously reported that self‐assembly of perylene bisimide dyes, appended with oligoethylene glycol chains, is driven by entropic factors in water.18a While mechanistic studies on the self‐assembly process were hampered for this derivative due to its strong aggregation tendency, recently we succeeded in obtaining an understanding of the entropically driven self‐assembly for amphiphilic dyes in water utilizing smaller naphthalene bisimide homologues.18e During these investigations, we came across a surprising observation that the glycol chains in these molecules are back‐folded to the aromatic core to sequester the hydrophobic surfaces from the surrounding bulk water. We reasoned that this specific orientation might be crucial in directing the self‐assembly toward an entropic driven process. If this hypothesis is correct, molecules without this specific orientation of glycol chains should accordingly self‐assemble in water driven by enthalpic factors as in the organic solvent. To gain deeper understanding of these processes and to realize control over the enthalpically and entropically driven self‐assembly in water, we designed an array of bolaamphiphilic rylene bisimide dyes where the orientation of glycol chains was modified by a subtle change in the monomer design. **PBI 1** and **NBI 1**, designed analogous to our previous molecules and an example of Ghosh et al.,^21^ consists of a perylene and naphthalene bisimide core, respectively, and are appended on both sides with a phenyl substituent bearing three oligoethylene glycol (OEG) chains (henceforth referred to as wedge) (Figure 1 b). In contrast, for **PBI 2** and **NBI 2**, this amphiphilic wedge is attached via a methylene spacer to disfavor the back folding. In organic solvents, all the newly designed dyes self‐assemble at lower temperatures and disassemble at elevated temperatures, characteristic of an enthalpically driven self‐assembly process. Interestingly, in water, **PBI 1** and **NBI 1** follow an entropically driven self‐assembly which is favored at higher temperatures, whilst **PBI 2** and **NBI 2** show common enthalpically driven self‐assembly, favored at lower temperatures. Combined utilization of UV/Vis spectroscopy and isothermal titration calorimetry (ITC) allowed us to gain deeper insights into the thermodynamic properties pertaining to their self‐assembly. Structural insights obtained by 2D NMR and PM7 optimizations point towards the crucial role of the orientation of glycol chains in orchestrating such bias of self‐assembly in water.

## Results and Discussion

### Temperature‐dependent self‐assembly and morphology of the aggregates

The thermodynamic fingerprint of bolaamphiphilic PBIs and NBIs were first probed by temperature‐dependent UV/Vis measurements. Generally, moieties which self‐assemble in an entropically driven fashion show an increased aggregation tendency at higher temperatures and undergo disaggregation at lower temperatures. The reverse is true for enthalpically driven self‐assembly, where the aggregation is favored at lower temperatures. First, we investigated the temperature‐dependent changes of all the molecules in an organic solvent. In a solvent of intermediate polarity like CHCl_3_, both **PBI 1** and **PBI 2** exist like many other perylene bisimides with solubilizing groups at imide positions in their monomeric form^22^ in a wide concentration range and exhibit typical vibronic progressions of the S_0_→S_1_ electronic transition (Figure S1a,b). However, in a polar solvent like MeOH, both PBIs self‐assemble into supramolecular aggregates, owing to the stabilization by dispersion interactions originating from π‐π stacking.^22^ At lower temperatures, the transition corresponding to the 0–1 vibronic progression at 491 nm is prominent for **PBI 1** compared to that at 527 nm (0–0 transition), suggesting the formation of an H‐type aggregate (Figure S2a).^22^ However, with an increase in temperature, the ratio of the 0–0/0–1 transition intensity increases along with a concomitant hyperchromism, suggesting disaggregation. Similarly, temperature‐dependent measurements of **PBI 2** in MeOH also revealed the formation of H‐type aggregate at lower temperatures, which disassembles and regains the vibronic progression of the monomer at higher temperatures (Figure S2b).

Akin to the PBIs mentioned above, naphthalene bisimide derivatives **NBI 1** and **NBI 2** also exist in monomeric form in CHCl_3_ (Figure S1 c,d) and undergo self‐assembly in MeOH at lower temperatures (Figure S2 c,d). However, the propensity of aggregation is much lower compared to their PBI homologues attributed to a less extended π‐core.^23^ Here also, the H‐type aggregates formed upon cooling disassemble at elevated temperatures, indicated by the hyperchromism along with the reinstating of monomer vibronic structure. In a gist, all the derivatives presented in the current work undergo an “ordinary temperature response” in MeOH, where the aggregation is favored at lower temperatures, driven by enthalpic factors.

**Figure 2:**
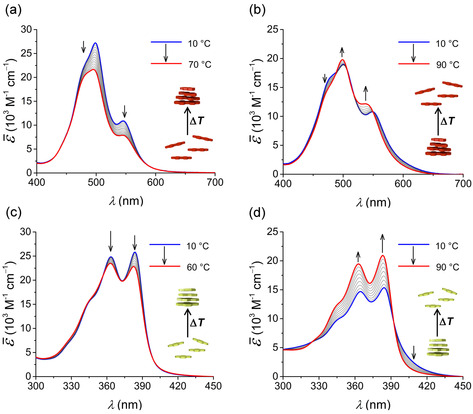
Temperature‐dependent UV/Vis spectra (density corrected) of (a) **PBI 1** (*c=*9.9×10^−6^ m), (b) **PBI 2** (*c=*9.8×10^−6^ m), (c) **NBI 1** (*c=*3.9×10^−5^ m) and (d) **NBI 2** (*c=*1.0×10^−4^ m) in water. Arrows indicate spectral changes upon heating.

Subsequently, we have investigated the temperature‐dependent self‐assembly of all the derivatives in water. **PBI 1**, even at very low concentrations, exists in an aggregated state in water with a prominent 0–1 transition (at 498 nm) suggesting a co‐facial packing of the molecules with an H‐type excitonic coupling (Figure 2 a). Surprisingly, upon increasing the temperature, we observed a pronounced hypochromic shift for the bands at 498 nm and 545 nm along with a decrease in the ratio of 0–0/0–1 transitions, opposite to its behavior in MeOH. These spectral changes point towards an increased degree of association, that is, aggregate growth into larger structures as reported before for an analogous PBI derivative in water.18a Similarly, **NBI 1**, which exhibits a vibronic progression of its monomeric state at lower temperatures, undergoes aggregate growth at elevated temperatures as indicated by hypochromism along with a concomitant decrease in the ratio of the vibronic transition at 384 nm with respect to that at 363 nm (Figure 2 c). In both cases, spectral changes upon temperature variation in water are contrary to the behavior in MeOH and point towards an increasing tendency of association with temperature, indicating the contribution of entropic factors in driving the self‐assembly.

**PBI 2**, in which the amphiphilic wedge is attached via a CH_2_ spacer, also exhibits H‐type aggregation due to co‐facial stacking in water (Figure 2 b). However, unlike **PBI 1**, here we observed the emergence of the 0–0 vibronic transition of the monomer band at 537 nm upon increasing temperature, suggesting a partial disassembly of the aggregate. In an even more pronounced fashion, **NBI 2** also disassembles at higher temperatures as indicated by hyperchromism and monomer‐like vibronic progression, identical to its behavior in MeOH (Figure 2 d). Both these observations indicate the ‘ordinary temperature response’ exhibited by **PBI 2** and **NBI 2** in water, where the aggregate formation is disfavored at higher temperatures.

Unfortunately, quantitative insights into the thermodynamic parameters of the self‐assembly are difficult to derive from these temperature‐dependent UV/Vis measurements since the fraction of aggregated species covered in the accessible temperature window remained narrow. Nevertheless, these experiments reveal a surprising bias of self‐assembly in water, where bolaamphiphilic molecules with amphiphilic wedge directly connected to the core undergo an entropically driven self‐assembly, favored at higher temperatures, whereas those having the wedge connected through a methylene spacer undergo enthalpically driven self‐assembly, like in organic solvents.

To obtain insights into the morphology of aggregates in water, atomic force microscopy (AFM) measurements were performed on all the derivatives by spin coating aqueous stock solutions at 22 °C (Figure S3). Predominantly, island‐like clusters were observed for **PBI 1**, **PBI 2** and **NBI 1** with height profiles ranging from 1.0–1.8 nm. Closer inspection revealed that these lamellar structures are formed by the bundling of one dimensional nanofibers, which might be due to the interdigitation of long side chains at higher concentrations. For **NBI 2**, we observed shorter nanoparticles with a height of 1.5–1.8 nm.

### Thermodynamic profiling of self‐assembly

Intrigued by the unique bias of the self‐assembly in water by subtle substituent variation, we decided to obtain deeper insights into the parameters which pertain the association as well as their self‐assembly mechanism in this solvent. For this, we applied concentration‐dependent UV/Vis studies as an effective tool since a broader range of aggregated species can be covered, surpassing the limitations of our previous experiments. Initially, we measured the UV/Vis spectra of **PBI 1** and **PBI 2** at different concentrations in water at 25 °C (Figure S4). However, very little changes in their vibronic structure were observed with varying concentrations owing to the high aggregation tendency of these extended π‐scaffolds in water. The naphthalene derivatives **NBI 1** and **NBI 2** appeared to be suitable candidates in this respect since their smaller π‐core assert moderate aggregation constants in aqueous media.

**Figure 3:**
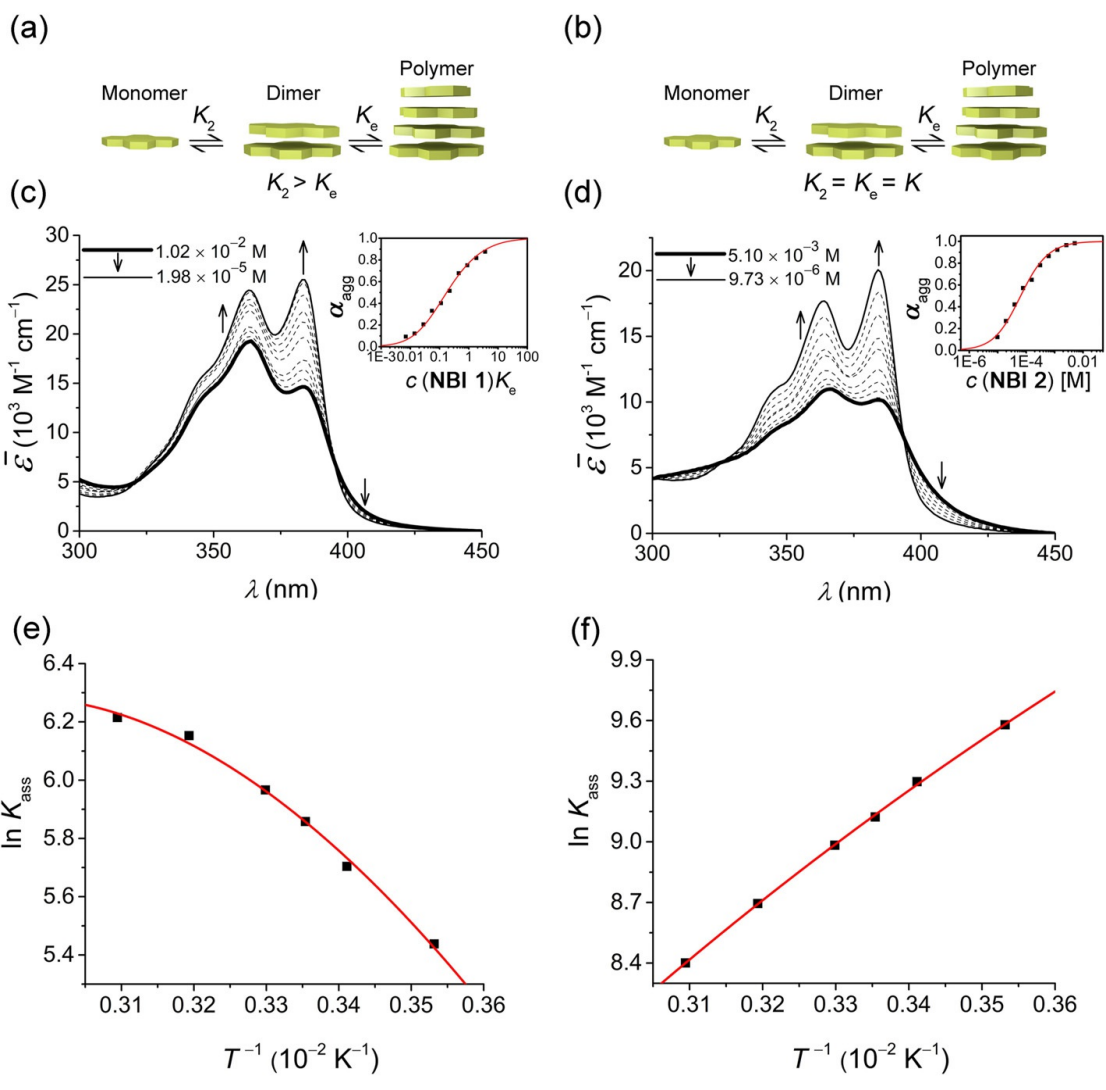
Schematic diagram showing self‐assembly via (a) anti‐cooperative and (b) isodesmic mechanism. (c) Concentration‐dependent UV/Vis spectra of **NBI 1** in water at 25 °C. Inset: Corresponding plot of the fraction of aggregated species, *α*_agg_, against dimensionless product *c* (**NBI 1**)*K*_e_ and analysis of the data based on the Goldstein–Stryer model. (d) Concentration‐dependent UV/Vis spectra of **NBI 2** in water at 25 °C. Inset: Corresponding plot of the fraction of aggregated species, *α*_agg_, against concentration and analysis of the data based on the isodesmic model. Plot of natural logarithm of association constant (ln *K*_ass_) against reciprocal of temperature and corresponding fit according to Clarke–Glew equation for (e) **NBI 1** and (f) **NBI 2**.

At lower concentrations, **NBI 1** in water shows distinct vibronic progression akin to monomeric spectra with an absorption maximum, *λ*_max_, at 384 nm, corresponding to the 0–0 transition (Figure 3 c). With an increase in concentration, we could observe a hypochromic shift along with the absorption maximum shifting to the 0–1 transition at 363 nm, correlating to the spectral changes observed in temperature‐dependent measurements. Such a pattern is characteristic of an H‐type excitonic coupling, where the π‐surfaces are arranged co‐facially in an aggregate. While plotting the corresponding degree of aggregation against the dimensionless product *c* (**NBI 1**)*K*_e_, we noticed a deviation from the sigmoidal transition characteristic for the isodesmic self‐assembly. An analysis by Goldstein‐Stryer model^24^ according to the Equation (1) revealed that **NBI 1** aggregates in a weakly anti‐cooperative fashion with a cooperativity factor *σ*=5 and a nucleus size of 2 (for details, see the Supporting Information).

(1) $$K_{e}c_{T}=\sum_{n-1}^{s}n\sigma^{n-1}{\left(K_{e}c_{1}\right)}^{n}+\sum_{n=s+1}^{\infty }n\sigma^{s-1}{\left(K_{e}c_{1}\right)}^{n}$$

in which *K*_e_ is the elongation constant, *s* is the nucleus size, *σ* is the cooperativity factor, *c*_1_ is the monomeric concentration and $c_{T}$ , the total concentration of dye molecules.

In such processes, the nucleation is more favored than the elongation toward extended aggregates. It is likely that this anti‐cooperativity arises from the sterical congestion imparted by the oligoethylene glycol wedges upon self‐assembly in a discotic stack. Noticeably, the association constant of elongation regime in water is quite weak (*K*_e_=350 m ^−1^ at 25 °C) considering the strong hydrophobic interactions operative in this medium.

Similarly, for **NBI 2** concentration‐dependent studies were performed in water at 25 °C (Figure 3 d). With increasing concentration, *λ*_max_ at 384 nm is shifted to 365 nm, implying the formation of an H‐type aggregate as in the previous case. However, unlike **NBI 1**, the transition from monomers to aggregate trace a sigmoidal shape within this concentration regime explained well by the isodesmic model according to Equation (2).^23^

(2) $$\alpha_{agg}=1-\frac{2Kc_{T}+1-\sqrt{4Kc_{T}+1}}{2K^{2}c_{T}^{2}}$$

in which $\alpha_{agg}\;$ is the degree of aggregation, *K* is the aggregation constant and $c_{T}$ , the total concentration of molecules.

Even though the two derivatives differ only by a CH_2_ group in imide substituents, the aggregation constant of **NBI 2** (*K=*9.1×10^3^ m ^−1^ at 25 °C) is 25‐fold higher than that of **NBI 1**. Such an increase in binding strength is unlikely to be caused by the hydrophobic nature of the CH_2_ group alone and might reside in the specific geometry of the amphiphilic wedges.

In an attempt to quantify the thermodynamic parameters encoded in the self‐assembly of **NBI 1** and **NBI 2**, we performed these concentration‐dependent experiments at different temperatures from 10 °C to 50 °C (Figures S5, S6). The corresponding natural logarithms of the association constants were then plotted against the inverse of temperature as shown in Figure 3 e, f. The association constants of **NBI 1** indeed increases with rising temperatures (Figure 3 e), which corroborates with our temperature dependent UV/Vis measurements. Furthermore, **NBI 1** reveals a non‐linear relationship between the association constants with respect to temperature. The traditional van't Hoff plot is inadequate to describe such systems which deviate from linearity due to the assumption of constant enthalpy and entropy at different temperatures.^25^ Hence, we utilized a simplified Clarke–Glew Equation (3) (also referred to as integrated/extended van't Hoff equation) by incorporating the change in heat capacity at constant pressure, $\Delta C_{p}$ .18e, 26 According to this, the natural logarithm of association constant at temperature *T* is expressed as:

(3) $$\;\ln\;\left[K\left(T\right)\right]=\ln\;\left[K\left(\theta \right)\right]+\frac{\Delta H\left(\theta \right)}{R}\left[\frac{1}{\theta }-\frac{1}{T}\right]+\frac{\Delta C_{p}\left(\theta \right)}{R}\left[\frac{\theta }{T}-1+\ln\left(\frac{T}{\theta }\right)\right]$$

in which, $\ln\;\left[K\left(\theta \right)\right]\;$ is the natural logarithm of the equilibrium constant at the reference temperature θ , $\Delta H\left(\theta \right)$ is the enthalpy change at the reference temperature and $\Delta C_{p}$ is the change in heat capacity at constant pressure.

Indeed, the present method provides a better fit for the variation of association constants with temperature. Such a curve exhibits a negative slope, suggesting that the self‐assembly process is endothermic (enthalpically disfavoured). The standard enthalpy change of association, Δ*H*°_ass_, of 17.0 kJ mol^−1^ derived from the Clarke–Glew equation indeed supports this observation. However, the entropic component, −*T*Δ*S*°_ass_ occupies a value of −31.5 kJ mol^−1^, making the overall process spontaneous with a Δ*G*°_ass_ of −14.5 kJ mol^−1^. Furthermore, Clarke–Glew equation also allows us to calculate a heat capacity change at a constant pressure of −419 J mol^−1^ K^−1^ for **NBI 1**. The endothermic nature of **NBI 1** self‐assembly in water is further confirmed by an independent method, i.e., isothermal titration calorimetry (ITC). In order to trace the heat signals associated with the self‐assembly of current derivatives, we performed an ITC dilution experiment where a concentrated aqueous solution of the corresponding molecule was injected into pure water taken in the cell.^27^ While performing this, we observed that **NBI 1** gives exothermic signals upon dilution (or dissociation) confirming that the association process is endothermic (enthalpically disfavored) (Figure S7a,b).

**Figure 4:**
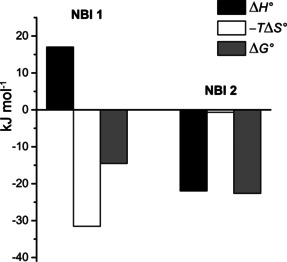
Thermodynamic profiles for the self‐assembly of **NBI 1** and **NBI 2** in water obtained by concentration‐dependent UV/Vis experiments.

**NBI 2** exhibits a linear relationship between the logarithm of its association constant and the reciprocal of temperature, and in contrast to **NBI 1**, a decrease in association constants was observed with elevated temperatures (Figure 3 f). The positive slope of the curve suggests an exothermic self‐assembly process, which is enthalpically favoured. A standard enthalpy change, Δ*H*°_ass_, of −21.9 kJ mol^−1^ and a standard entropy change, −*T*Δ*S*°_ass_, of −0.6 kJ mol^−1^ is deduced for the self‐assembly of **NBI 2** from Clarke–Glew equation. Agreeing with our previous observations of a higher aggregation tendency for **NBI 2**, the change in standard free energy, Δ*G*°_ass_, possesses a value of −22.6 kJ mol^−1^, which is considerably lower than that of **NBI 1**. Finally, a heat capacity change at constant pressure, $\Delta C_{p}$ , of −128 J mol^−1^ K^−1^ is estimated for the self‐assembly of **NBI 2**. ITC dilution experiment was conducted also here to verify the exothermic nature of **NBI 2** self‐assembly (Figure S7c,d). The dilution of **NBI 2** aggregate initially gave endothermic signals (corresponding to disassembly), which subsided after a few injections while reaching saturation, and showed constant exothermic signals corresponding to the heat of injection. This supports our observation from UV/Vis experiments that the self‐assembly of **NBI 2** is enthalpy driven. The combined thermodynamic signature for **NBI 1** and **NBI 2** aggregation at 25 °C is depicted in Figure 4.

### Structural insights by 2D NMR and PM7 calculations

In order to obtain structural insights to explain the striking difference in the self‐assembly characteristics of the two molecules with and without a methylene spacer, we resorted to 1D and 2D NMR studies. The ^1^H NMR of **NBI 1** in CDCl_3_ shows sharp and well‐resolved signals, suggesting a monomeric state (Figure S8). However, in D_2_O the naphthalene core protons are upfield shifted and broadened suggesting a self‐assembled state aided by π–π stacking. In **NBI 2**, the naphthalene protons are more upfield shifted and broadened in comparison to **NBI 1** at the same concentration (Figure S9). This corroborates with the higher aggregation tendency observed for **NBI 2** in our UV/Vis studies.

**Figure 5:**
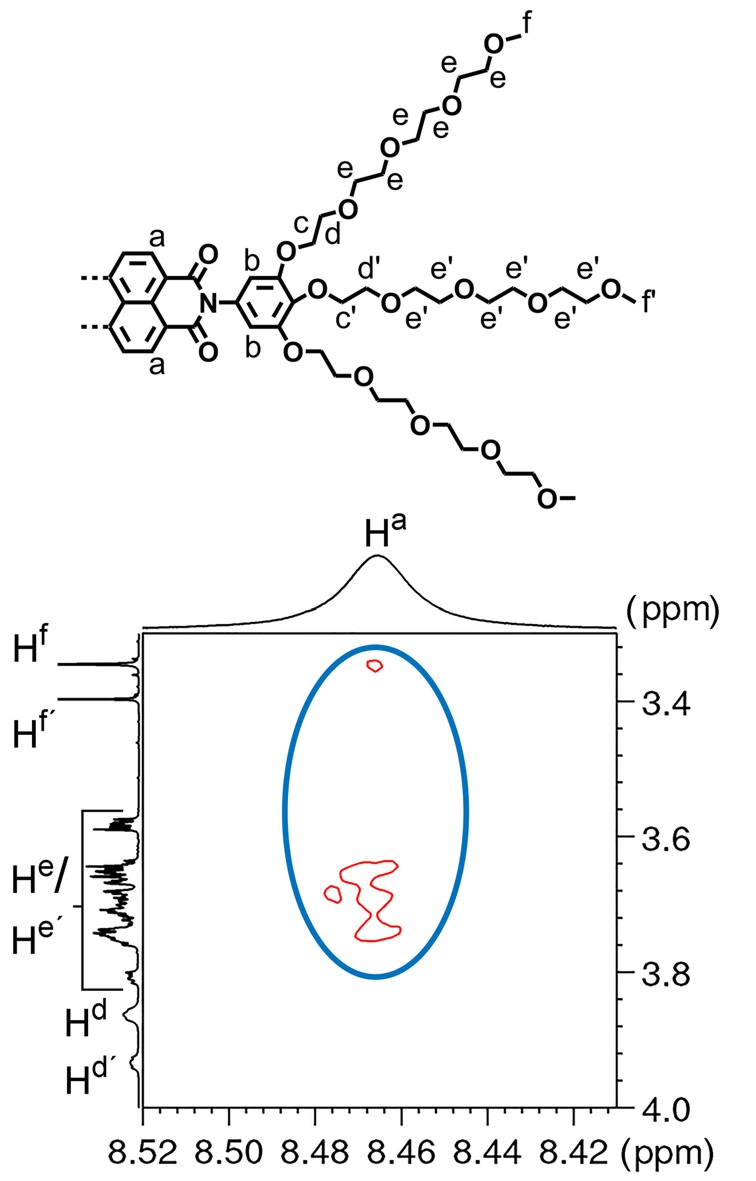
Partial chemical structure of **NBI 1** with significant protons assigned (above). Selected region of ^1^H‐^1^H ROESY spectrum of **NBI 1** in D_2_O (*c=*5.0×10^−3^ m) showing NOEs between glycol chains and naphthalene core (circle).

Subsequently, the discrepancies between the aggregate structure of both compounds were probed via ^1^H–^1^H rotating‐frame Overhauser effect spectroscopy (ROESY). In Figure 5, a selected region of ROESY spectrum of **NBI 1** in D_2_O is shown. Interestingly, nuclear Overhauser effect (NOE) correlations could be observed between the glycol chain protons (H^e^/H^e’^, H^f^) and naphthalene core protons (H^a^) of the **NBI 1**, indicating that the OEG chains are proximal to the hydrophobic core. Such a correlation can only be explained if the chains are back‐folded over the aromatic core and not extended into the bulk solvent. Recently, we have observed such a folding process on analogous naphthalene bisimide derivatives in molecular dynamics (MD) and 2D NMR studies.18e Previously, other groups have attributed this conformation of side chains to the shielding of the π‐surface due to hydrophobic effects.9b, 28 It should be noted that this process is exclusive to aqueous media and was not observed for the ROESY studies of **NBI 1** in an organic solvent (Figure S10). Cross peaks corresponding to such a folding process could not be observed in the ROESY spectrum of **NBI 2** in D_2_O (Figure S11).

In an effort to understand what leads to the proximity of these chains to the core in the case of **NBI 1** and not in **NBI 2**, we resorted to semi‐empirical PM7 calculations in water (Figure S12). The optimized geometry of **NBI 1** in water shows that the phenyl ring of the amphiphilic wedge is oriented nearly coplanar to the naphthalene core. This facilitates the OEG chains to fold backwards to sequester the hydrophobic component from the bulk water. In contrast, the amphiphilic wedge is oriented almost orthogonal to the naphthalene core in **NBI 2** imparted by the sp^3^ hybridization of the CH_2_ spacer group. As a result, the glycol chains in **NBI 2** are extended away from the π‐core, and thus their propensity to fold backwards is limited compared to that of **NBI 1**.

### Interpretation of the enthalpy versus entropy driven aggregation

**Figure 6:**
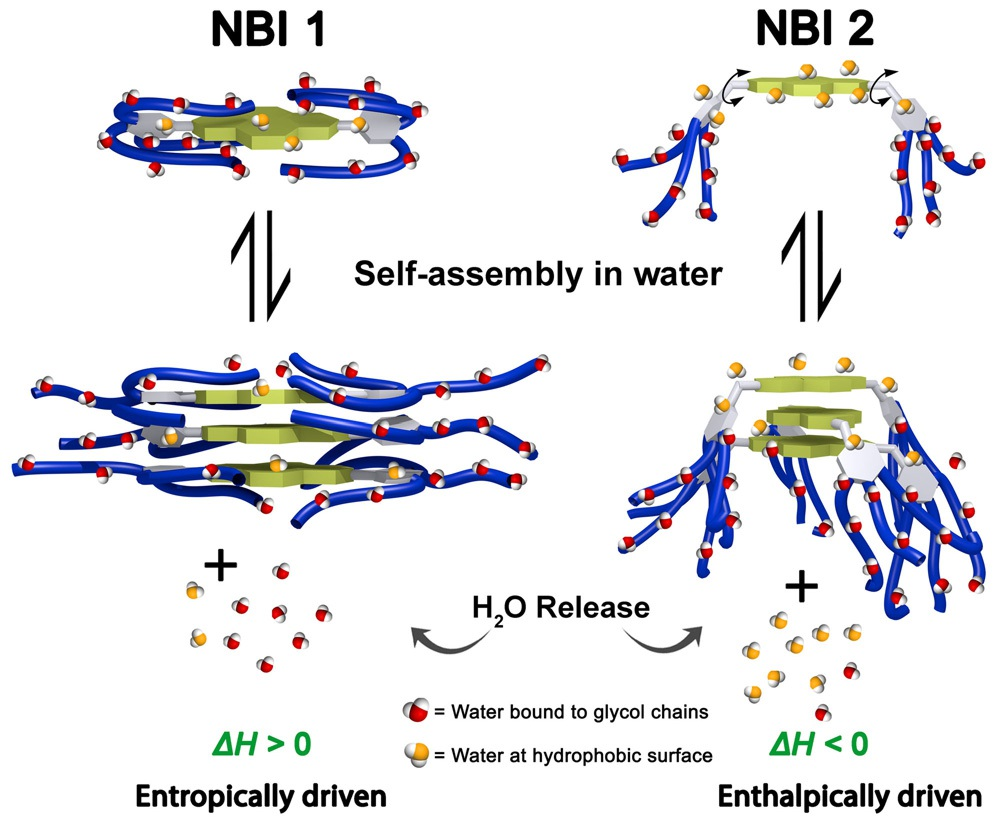
Schematic representation of self‐assembly processes of **NBI 1** (left) and **NBI 2** (right) in water. At glycol chains, H‐bonded water molecules are indicated in red, while the “high‐energy” water molecules at hydrophobic surfaces are shown in orange. The double‐headed arrows in **NBI 2** structure represents possible rotation of the wedges.

The common explanation for entropy driven aggregation processes as observed for **NBI 1** and **PBI 1** is a release of hydrated H_2_O molecules forming H‐bonds with the glycol units (Figure 6).^14^, ^29^ This explanation is reasonable and has been adopted in our previous work18a, 18d, 18e because sterically demanding wedges as given with OEG side chains cannot accommodate larger amounts of coordinated water molecules without sterical encumbrance upon close cofacial stacking of the dyes. Accordingly, a significant amount of these glycol‐bound water molecules have to be released to aid the association. The energy‐intensive process of breaking these H‐bonds surpasses the enthalpic gain arising from π–π stacking, making the overall enthalpy of aggregation positive. However, removal of hydrated water molecules into the bulk solvent leads to a surge in the entropy, which drives the self‐assembly.

However, taking into account the structural characterizations and the differences in the thermodynamic signature for the aggregation processes of the here investigated dyes, we propose that another effect plays a major role and that the aggregation processes of **NBI 1** and **PBI 1** are primarily governed by the gain in entropy originating from the release of the back‐folded OEG chains. Indeed, to sequester the hydrophobic π‐surfaces of these rylene dyes a large number of otherwise freely rotating bonds have to be frozen in a specific conformation. The concomitant entropic loss by freezing such a multitude of conformationally mobile sections has to be huge and accordingly the release of OEG chains upon π–π stacking of the dyes will provide a huge gain in entropy.^30^ This view is also supported by the quite low aggregation constant determined by UV/Vis experiments for **NBI 1** considering the solvophobic effects operative in water. Back‐folding of glycol chains restricts the π–π stacking due to steric hindrance, which in turn decreases the aggregation constant. Furthermore, we observed that the mechanism of aggregation of **NBI 1** deviates from the isodesmic model and is better described by a weakly anti‐cooperative model. The anti‐cooperativity observed for **NBI 1** can also be attributed to such sterical constraint, which is aided by the back‐folding process as observed in our ROESY experiments.

Our results might be also of relevance for biological systems, for which the entropically driven host‐guest binding and self‐assembly is in general attributed to an increase in entropy of water molecules upon release into the bulk.^31^ Here also back‐folded side chains might often be redistributed more flexibly upon aggregation, which could contribute to the overall entropy via an increase in the conformational entropy of the chains.

In contrast to these dyes, the thermodynamic signatures of **NBI 2** and **PBI 2** reveal that the association of these molecules is primarily driven by enthalpic factors. The lack of spatial correlations of the side chains and the π‐core in 2D NMR studies of **NBI 2** indicates that the hydration shells of glycol chains are mostly arranged in an extended fashion away from the rylene core (Figure 6). Thus, during the self‐assembly of monomers by π–π stacking there is no significant increase in the degrees of freedom of side chains upon aggregation due to the lack of back‐folding. This, in turn, means that the thermodynamic signature is primarily arising from the contributions of the hydrophobic NBI core, which probably stacks co‐facially in a cogwheel type arrangement as reported for a similar derivative by Percec et al.^32^ The water molecules at such non‐polar surfaces are known to be energetically frustrated due to the less‐favored H‐bonding capacity with respect to bulk water and are termed as “high energy water”.^33^ Upon association, these water molecules are released into the bulk, leading to a large energetic gain that drives the self‐assembly via favorable enthalpy, leading to a non‐classical hydrophobic interaction. Furthermore, dispersion forces from the π–π interactions might also contribute to the favorable stacking.^34^ Unlike **NBI 1**, the π‐stacking of **NBI 2** is not counteracted by the folded chains since they are extended away from the core. This leads to a higher association constant as revealed by concentration‐dependent studies. Furthermore, the lack of sterical constraints for the more flexibly attached OEG wedges (due to a rotatable methylene group) allows **NBI 2** to aggregate in an isodesmic manner.

## Conclusions

The understanding of the relevant thermodynamic parameters is quintessential to control self‐assembly processes. In this regard, we demonstrated here the importance of conformational effects with a subtle modification, that is, addition of a simple CH_2_ spacer unit, to modulate an aggregation process from enthalpically to entropically driven for archetypal rylene bisimide dyes in water. In an organic solvent like methanol, all the studied molecules aggregate upon cooling and dissociate upon heating. In water, however, molecules in which the amphiphilic wedge is directly attached to the π‐core (**PBI 1** and **NBI 1**) exhibit entropically driven self‐assembly where the aggregation is favoured at higher temperatures. In contrast, compounds where the wedge is attached via a methylene spacer (**PBI 2** and **NBI 2**) aggregate upon cooling driven by enthalpic factors. In‐depth concentration‐ and temperature‐dependent UV/Vis studies allowed us to extract the thermodynamic parameters of their self‐assembly, which was further confirmed by ITC measurements. Structural insights obtained by 2D NMR and PM7 studies point towards the role of the orientation of side chains in orchestrating this bifurcated thermal response. We hypothesize that many if not all amphiphilic systems characterized by such back folded conformations in water will aggregate in an entropy‐driven fashion due to the substantial gain in entropy upon liberation of conformationally frozen rotatable hydrophilic side chains. Therefore, the elucidation of the temperature‐dependent self‐assembly of other π‐amphiphiles in water will be of high interest. Further, we suggest considering this phenomenon also for supramolecular interactions involving biomacromolecules.

## Conflict of interest

The authors declare no conflict of interest.

## Supporting information

As a service to our authors and readers, this journal provides supporting information supplied by the authors. Such materials are peer reviewed and may be re‐organized for online delivery, but are not copy‐edited or typeset. Technical support issues arising from supporting information (other than missing files) should be addressed to the authors.

Supplementary

## References

[1a] T. Aida , E. W. Meijer , S. I. Stupp , Science 2012, 335, 813–817; 22344437 10.1126/science.1205962 PMC3291483

[1b] T. F. A. De Greef , M. M. J. Smulders , M. Wolffs , A. P. H. J. Schenning , R. P. Sijbesma , E. W. Meijer , Chem. Rev. 2009, 109, 5687–5754; 19769364 10.1021/cr900181u

[1c] X. Yan , F. Wang , B. Zheng , F. Huang , Chem. Soc. Rev. 2012, 41, 6042–6065. 22618080 10.1039/c2cs35091b

[2] F. Tantakitti , J. Boekhoven , X. Wang , R. V. Kazantsev , T. Yu , J. Li , E. Zhuang , R. Zandi , J. H. Ortony , C. J. Newcomb , L. C. Palmer , G. S. Shekhawat , M. O. de la Cruz , G. C. Schatz , S. I. Stupp , Nat. Mater. 2016, 15, 469–476. 26779883 10.1038/nmat4538 PMC4805452

[3a] E. Krieg , M. M. C. Bastings , P. Besenius , B. Rybtchinski , Chem. Rev. 2016, 116, 2414–2477; 26727633 10.1021/acs.chemrev.5b00369

[3b] D. Görl , X. Zhang , F. Würthner , Angew. Chem. Int. Ed. 2012, 51, 6328–6348; 10.1002/anie.201108690 22573415

[3c] B. N. S. Thota , L. H. Urner , R. Haag , Chem. Rev. 2016, 116, 2079–2102. 26669418 10.1021/acs.chemrev.5b00417

[4] N. J. Van Zee , B. Adelizzi , M. F. J. Mabesoone , X. Meng , A. Aloi , R. H. Zha , M. Lutz , I. A. W. Filot , A. R. A. Palmans , E. W. Meijer , Nature 2018, 558, 100–103. 29849144 10.1038/s41586-018-0169-0

[5a] B. Kemper , L. Zengerling , D. Spitzer , R. Otter , T. Bauer , P. Besenius , J. Am. Chem. Soc. 2018, 140, 534–537; 29271649 10.1021/jacs.7b08189

[5b] M. P. Hendricks , K. Sato , L. C. Palmer , S. I. Stupp , Acc. Chem. Res. 2017, 50, 2440–2448. 28876055 10.1021/acs.accounts.7b00297 PMC5647873

[6a] K. V. Rao , S. J. George , Org. Lett. 2010, 12, 2656–2659; 20450161 10.1021/ol100864e

[6b] J. P. Hill , W. Jin , A. Kosaka , T. Fukushima , H. Ichihara , T. Shimomura , K. Ito , T. Hashizume , N. Ishii , T. Aida , Science 2004, 304, 1481–1483; 15178796 10.1126/science.1097789

[6c] M. Yin , J. Shen , W. Pisula , M. Liang , L. Zhi , K. Müllen , J. Am. Chem. Soc. 2009, 131, 14618–14619. 19824720 10.1021/ja9058662

[7a] M. R. Molla , S. Ghosh , Phys. Chem. Chem. Phys. 2014, 16, 26672–26683; 25375094 10.1039/c4cp03791j

[7b] A. Sikder , D. Ray , V. K. Aswal , S. Ghosh , Angew. Chem. Int. Ed. 2019, 58, 1606–1611; 10.1002/anie.201812217 30421845

[7c] M. Al Kobaisi , S. V. Bhosale , K. Latham , A. M. Raynor , S. V. Bhosale , Chem. Rev. 2016, 116, 11685–11796. 27564253 10.1021/acs.chemrev.6b00160

[8a] M. Sun , K. Müllen , M. Yin , Chem. Soc. Rev. 2016, 45, 1513–1528; 26797049 10.1039/c5cs00754b

[8b] D. Görl , X. Zhang , V. Stepanenko , F. Würthner , Nat. Commun. 2015, 6, 7009–7017; 25959777 10.1038/ncomms8009 PMC4432616

[8c] M. Ogasawara , X. Lin , H. Kurata , H. Ouchi , M. Yamauchi , T. Ohba , T. Kajitani , T. Fukushima , M. Numata , R. Nogami , B. Adhikari , S. Yagai , Mater. Chem. Front. 2018, 2, 171–179;

[8d] A. Ustinov , H. Weissman , E. Shirman , I. Pinkas , X. Zuo , B. Rybtchinski , J. Am. Chem. Soc. 2011, 133, 16201–16211; 21882828 10.1021/ja2066225

[8e] M. Hariharan , Y. Zheng , H. Long , T. A. Zeidan , G. C. Schatz , J. Vura-Weis , M. R. Wasielewski , X. Zuo , D. M. Tiede , F. D. Lewis , J. Am. Chem. Soc. 2009, 131, 5920–5929. 19382814 10.1021/ja900347t

[9a] N. M. Matsumoto , R. P. M. Lafleur , X. Lou , K.-C. Shih , S. P. W. Wijnands , C. Guibert , J. W. A. M. van Rosendaal , I. K. Voets , A. R. A. Palmans , Y. Lin , E. W. Meijer , J. Am. Chem. Soc. 2018, 140, 13308–13316; 30221520 10.1021/jacs.8b07697 PMC6194755

[9b] M. B. Baker , L. Albertazzi , I. K. Voets , C. M. Leenders , A. R. A. Palmans , G. M. Pavan , E. W. Meijer , Nat. Commun. 2015, 6, 6234–6246; 25698667 10.1038/ncomms7234 PMC4346625

[9c] P. Besenius , G. Portale , P. H. H. Bomans , H. M. Janssen , A. R. A. Palmans , E. W. Meijer , Proc. Natl. Acad. Sci. USA 2010, 107, 17888–17893. 20921365 10.1073/pnas.1009592107 PMC2964246

[10a] Y. Kim , T. Kim , M. Lee , Polym. Chem. 2013, 4, 1300–1308;

[10b] Z. Huang , S.-K. Kang , M. Banno , T. Yamaguchi , D. Lee , C. Seok , E. Yashima , M. Lee , Science 2012, 337, 1521–1526. 22997334 10.1126/science.1224741

[11a] C. Rest , M. J. Mayoral , K. Fucke , J. Schellheimer , V. Stepanenko , G. Fernández , Angew. Chem. Int. Ed. 2014, 53, 700–705; 10.1002/anie.201307806 24352814

[11b] T. Rudolph , N. Kumar Allampally , G. Fernández , F. H. Schacher , Chem. Eur. J. 2014, 20, 13871–13875. 25201355 10.1002/chem.201404141

[12a] R. Thirumalai , R. D. Mukhopadhyay , V. K. Praveen , A. Ajayaghosh , Sci. Rep. 2015, 5, 9842–9853; 25940779 10.1038/srep09842 PMC4419532

[12b] J. F. Hulvat , M. Sofos , K. Tajima , S. I. Stupp , J. Am. Chem. Soc. 2005, 127, 366–372. 15631487 10.1021/ja047210m

[13] P. S. Cremer , A. H. Flood , B. C. Gibb , D. L. Mobley , Nat. Chem. 2018, 10, 8–16. 10.1038/nchem.2894 29256514

[14] N. Saito , H. Kobayashi , M. Yamaguchi , Chem. Sci. 2016, 7, 3574–3580. 29997850 10.1039/c5sc04959h PMC6007355

[15] R. A. Shalaby , M. A. Lauffer , Arch. Biochem. Biophys. 1985, 236, 390–398. 3966803 10.1016/0003-9861(85)90639-3

[16] P. Friedhoff , A. Schneider , E.-M. Mandelkow , E. Mandelkow , Biochemistry 1998, 37, 10223–10230. 9665729 10.1021/bi980537d

[17] K. E. Kadler , Y. Hojima , D. J. Prockop , J. Biol. Chem. 1987, 262, 15696–15701. 3316206

[18a] D. Görl , F. Würthner , Angew. Chem. Int. Ed. 2016, 55, 12094–12098; 10.1002/anie.201606917 27558471

[18b] P. Dey , P. Rajdev , P. Pramanik , S. Ghosh , Macromolecules 2018, 51, 5182–5190;

[18c] H. Fenniri , B. L. Deng , A. E. Ribbe , K. Hallenga , J. Jacob , P. Thiyagarajan , Proc. Natl. Acad. Sci. USA 2002, 99, 6487–6492; 11891281 10.1073/pnas.032527099 PMC128555

[18d] D. Görl , B. Soberats , S. Herbst , V. Stepanenko , F. Würthner , Chem. Sci. 2016, 7, 6786–6790; 28451124 10.1039/c6sc02249a PMC5356028

[18e] P. P. N. Syamala , B. Soberats , D. Görl , S. Gekle , F. Würthner , Chem. Sci. 2019, 10, 9358–9366. 32110300 10.1039/c9sc03103k PMC7017873

[19a] G. G. Ferenczy , G. M. Keserű , Drug Discovery Today 2010, 15, 919–932; 20801227 10.1016/j.drudis.2010.08.013

[19b] S. Geschwindner , J. Ulander , P. Johansson , J. Med. Chem. 2015, 58, 6321–6335. 25915439 10.1021/jm501511f

[20] G. Klebe , Nat. Rev. Drug Discovery 2015, 14, 95–110. 25614222 10.1038/nrd4486

[21] P. Rajdev , M. R. Molla , S. Ghosh , Langmuir 2014, 30, 1969–1976. 24494820 10.1021/la500089b

[22] Z. Chen , B. Fimmel , F. Würthner , Org. Biomol. Chem. 2012, 10, 5845–5855. 22391667 10.1039/c2ob07131b

[23] Z. Chen , A. Lohr , C. R. Saha-Möller , F. Würthner , Chem. Soc. Rev. 2009, 38, 564–584. 19169466 10.1039/b809359h

[24a] R. F. Goldstein , L. Stryer , Biophys. J. 1986, 50, 583–599; 3779001 10.1016/S0006-3495(86)83498-1 PMC1329836

[24b] M. J. Mayoral , C. Rest , V. Stepanenko , J. Schellheimer , R. Q. Albuquerque , G. Fernández , J. Am. Chem. Soc. 2013, 135, 2148–2151. 23347358 10.1021/ja312628g

[25] H. Naghibi , A. Tamura , J. M. Sturtevant , Proc. Natl. Acad. Sci. USA 1995, 92, 5597–5599. 7777555 10.1073/pnas.92.12.5597 PMC41743

[26a] E. C. W. Clarke , D. N. Glew , Trans. Faraday Soc. 1966, 62, 539–547;

[26b] M. J. Blandamer , Chemical Equilibria in Solution, Ellis Horwood, Chichester, 1992.

[27a] F. Aparicio , F. García , L. Sánchez , Chem. Eur. J. 2013, 19, 3239–3248; 23345173 10.1002/chem.201202584

[27b] A. Arnaud , L. Bouteiller , Langmuir 2004, 20, 6858–6863; 15274596 10.1021/la049365d

[27c] I. Turcu , M. Mic , J. Phys. Chem. B 2013, 117, 9083–9093. 23837441 10.1021/jp403768x

[28] M. Garzoni , M. B. Baker , C. M. A. Leenders , I. K. Voets , L. Albertazzi , A. R. A. Palmans , E. W. Meijer , G. M. Pavan , J. Am. Chem. Soc. 2016, 138, 13985–13995. 27696835 10.1021/jacs.6b07530

[29] T. Shikata , M. Okuzono , N. Sugimoto , Macromolecules 2013, 46, 1956–1961.

[30] G. M. Whitesides , J. P. Mathias , C. T. Seto , Science 1991, 254, 1312–1319. 1962191 10.1126/science.1962191

[31a] R. Alberstein , Y. Suzuki , F. Paesani , F. A. Tezcan , Nat. Chem. 2018, 10, 732–739; 29713036 10.1038/s41557-018-0053-4 PMC6056010

[31b] H. Oshima , T. Hayashi , M. Kinoshita , Biophys. J. 2016, 110, 2496–2506; 27276267 10.1016/j.bpj.2016.05.006 PMC4906361

[31c] T. Hayashi , H. Oshima , T. Mashima , T. Nagata , M. Katahira , M. Kinoshita , Nucleic Acids Res. 2014, 42, 6861–6875; 24803670 10.1093/nar/gku382 PMC4066790

[31d] T. Hayashi , H. Oshima , S. Yasuda , M. Kinoshita , J. Phys. Chem. B 2015, 119, 14120–14129. 26421917 10.1021/acs.jpcb.5b08513

[32] C. Roche , H.-J. Sun , P. Leowanawat , F. Araoka , B. E. Partridge , M. Peterca , D. A. Wilson , M. E. Prendergast , P. A. Heiney , R. Graf , H. W. Spiess , X. Zeng , G. Ungar , V. Percec , Nat. Chem. 2016, 8, 80–89. 26673268 10.1038/nchem.2397

[33a] F. Biedermann , V. D. Uzunova , O. A. Scherman , W. M. Nau , A. De Simone , J. Am. Chem. Soc. 2012, 134, 15318–15323; 22881280 10.1021/ja303309e

[33b] F. Biedermann , W. M. Nau , H.-J. Schneider , Angew. Chem. Int. Ed. 2014, 53, 11158–11171; 10.1002/anie.201310958 25070083

[34] F. Biedermann , in Comprehensive Supramolecular Chemistry II, (Ed.: J. L. Atwood), Elsevier, Oxford, 2017.

