# Supplementary material for: Modulation of the Self‐Assembly of π‐Amphiphiles in Water from Enthalpy‐ to Entropy‐Driven by Enwrapping Substituents
Source: Chemistry. 2020 Jun 17;26(38):8426–34. doi: 10.1002/chem.202000995 (PMC7384034; doi:10.1002/chem.202000995)
Supplement: Supplementary file 1 — Supplementary [file CHEM-26-8426-s001.pdf]

Chemistry–A European Journal

Supporting Information

Modulation of the Self-Assembly of π-Amphiphiles in Water from Enthalpy- to Entropy-Driven by Enwrapping Substituents

Pradeep P. N. Syamala[a, b] and Frank Würthner*[a, b]

Table of Contents

1. Materials and methods S2
2. Determination of thermodynamic parameters S3
3. Synthesis and characterization S5
4. Monomer UV-vis spectra S11
5. Temperature-dependent UV-vis experiments S12
6. Atomic force microscopy (AFM) S13
7. Concentration-dependent UV-vis experiments S13
8. Isothermal titration calorimetry (ITC) S16
9. NMR studies S17
10. PM7 calculations S20
11. NMR spectra of **PBI 1**, **PBI 2** and **NBI 1**, **NBI 2** S21
12. References S25

1. Materials and methods

General: Reagents were purchased from commercial suppliers and used as received without further purification. Solvents were distilled and dried according to standard procedures. Gravity column chromatography was performed with commercial glass columns using silica gel 60M (particle size 0.04-0.063 mm) as stationary phase, whereas flash coloumn chromatrography was carried out with a PuriFlash 420 system from *Interchim* using commercially available silica gel columns from *Interchim* (PF-30SIHP-F0025). NMR spectra were recorded on an Avance III HD (400 MHz or 600 MHz) from Bruker at 295 K. The spectra were calibrated to the residual protic solvent peak and the chemical shifts (δ) are recorded in parts per million (ppm). Multiplicities for proton signals are abbreviated as s, d, t, and m for singlet, doublet, triplet, and multiplet, respectively. High-resolution mass spectra (HRMS) were recorded with microTOF focus (Bruker Daltonics, Germany).

UV-vis spectroscopy: UV-vis absorption spectra were recorded using a JASCO V-770 spectrophotometer. The measurements were carried out in quartz cuvettes using either spectroscopic grade chloroform and methanol, or deionized water prepared by water purification system PURELAB classic (ELGA, France). Temperature control was accomplished by JASCO PAC-743R Peltier systems. Extinction coefficients were calculated from Lambert-Beer's law and are density corrected in the case of temperature-dependent measurements.

Isothermal titration calorimetry (ITC): ITC measurements were performed using PEAQ-ITC (Malvern) for **NBI 1** and MicroCal VP-ITC (GE Healthcare, USA) for **NBI 2**. The stock solutions as well as solvent (water) was degassed by applying vacuum before the measurements. The temperature of the sample solutions and water was adjusted to respective temperature prior to the measurement using a MicroCal ThermoVac (GE Healthcare, USA). The enthalpograms were derived by integrating the raw heat signals.

Molecular modelling: Structural optimization of **NBI 1** and **NBI 2** was carried out using semi-empirical PM7 Hamiltonian[1] in MOPAC2016.[2] Solvent effects have been included implicitly using the COSMO model for water.[3]

Atomic force microscopy (AFM): AFM measurements were performed under ambient conditions using a Bruker Multimode 8 SPM system operating in tapping mode in air. Silica cantilevers (OMCL-AC200TS, Olympus) with a resonance frequency of ~150 kHz and a spring constant of ~10 N m^{-1} were used. The samples were prepared by spin-coating of aqueous solutions onto either mica (for **PBI 1 & PBI 2**) or silicon wafer (treated by Argon plasma) (for **NBI 1 & NBI 2**).

2. Determination of thermodynamic parameters

Isodesmic model

One dimensional aggregation is assumed to proceed *via* same binding constant and free energy of aggregation in isodesmic model for each monomer added to the growing chain.[4] The equilibrium between monomer and aggregate for such a system is described as

$$\mathrm{M} + \mathrm{M} \overset{K_2}{\leftrightarrows} \mathrm{M_2}$$

$$\mathrm{M_2} + \mathrm{M} \overset{K_3}{\leftrightarrows} \mathrm{M_3}$$

....

$$\mathrm{M_{n-1}} + \mathrm{M} \overset{K_n}{\leftrightarrows} \mathrm{M_n}$$

For isodesmic model, $K_2 = K_3 = \cdots K_n$.

The molar fraction of aggregated species (α_{agg}) for such a system is expressed as

$$\alpha_{\mathrm{agg}} = 1 - \frac{2Kc_{\mathrm{T}}+1-\sqrt{4Kc_{\mathrm{T}}+1}}{2K^2c_{\mathrm{T}}^2} \qquad (1)$$

where K is the aggregation constant and c_{T} is the total concentration of molecules. α_{agg} can be expressed in terms of apparent extinction coefficient at a given dye concentration ($\bar{\varepsilon}(c_{\mathrm{T}})$) as

$$\alpha_{\mathrm{agg}} = 1 - \frac{\bar{\varepsilon}(c_{\mathrm{T}})-\varepsilon_{\mathrm{agg}}}{\varepsilon_{\mathrm{mon}}-\varepsilon_{\mathrm{agg}}} \qquad (2)$$

where $\varepsilon_{\mathrm{mon}}$ and $\varepsilon_{\mathrm{agg}}$ are the extinction coefficients of the aggregated and monomeric species, respectively. Combining eqn. (1) and (2), the apparent extinction coefficient at a given dye concentration, $\bar{\varepsilon}(c_{\mathrm{T}})$, is denoted as

$$\bar{\varepsilon}(c_{\mathrm{T}}) = \varepsilon_{\mathrm{agg}} + (\varepsilon_{\mathrm{mon}} - \varepsilon_{\mathrm{agg}})\frac{2Kc_{\mathrm{T}}+1-\sqrt{4Kc_{\mathrm{T}}+1}}{2K^2c_{\mathrm{T}}^2} \qquad (3)$$

By fitting plot of $\bar{\varepsilon}(c_{\mathrm{T}})$ at a specific wavelength with respect to concentration, c_{T}, using eqn (3) corresponding binding constant at a particular temperature can be determined. The Gibbs free energy of association, ΔG_{ass}, is computed using the following equation:

$$\Delta G_{\mathrm{ass}} = -RT\ln K_{\mathrm{ass}} \qquad (4)$$

where R is the universal gas constant, T is the temperature and K_{ass} the association constant.

Goldstein-Stryer model

The anti-cooperativity process is described using the Goldstein-Stryer model, which combines the formation of a nucleus with size '*s*' and further aggregation into larger structures.[5] The cooperativity factor σ is defined as the ratio of binding constants for nucleus formation (K_s) and further elongation (K_e).

$$\sigma = \frac{K_\mathrm{s}}{K_\mathrm{e}} \qquad (5)$$

For $\sigma = 1$, the process is considered as isodesmic while for $\sigma < 1$ cooperative and $\sigma > 1$ anti-cooperative. An anti-cooperative process can be evaluated by the Goldstein-Stryer model using the following equation. [6]

$$K_\mathrm{e}c_\mathrm{T} = \sum_{n-1}^{s} n\sigma^{n-1}(K_\mathrm{e}c_1)^n + \sum_{n=s+1}^{\infty} n\sigma^{s-1}(K_\mathrm{e}c_1)^n = \frac{s(K_\mathrm{e}c_1)^s\sigma^{s-1}}{1-K_\mathrm{e}c_1} + \frac{(K_\mathrm{e}c_1)^{s+1}\sigma^{s-1}}{(1-K_\mathrm{e}c_1)^2} + \frac{K_\mathrm{e}c_1(s(\sigma K_\mathrm{e}c_1)^{s-1}-1)}{\sigma K_\mathrm{e}c_1-1} - \frac{s(K_\mathrm{e}c_1)^2\,((\sigma K_\mathrm{e}c_1)^{s-1}-1)}{(\sigma K_\mathrm{e}c_1-1)^2} \qquad (6)$$

where K_e is the elongation constant, σ is the cooperativity factor, c_1 is the monomeric concentration and c_T the total concentration of dye molecules.

The Gibbs free energy of association, ΔG_{ass}, is computed using the following equation:

$$\Delta G_\mathrm{ass} = -RT\ln K_\mathrm{e} \qquad (7)$$

where R is the universal gas constant, T is the temperature and K_e the elongation constant.

3. Synthesis and characterization

1. Synthesis of **PBI 1** and **NBI 1**

PBI 1 and **NBI 1** were prepared according to an analogous synthetic route reported by Ghosh *et al.* and is depicted in Scheme S1.[7] The precursor methyl 3,4,5-tris((2,5,8,11-tetraoxatridecan-13-yl)oxy)benzoate (**1**) was synthesized according to reported procedure.[8]

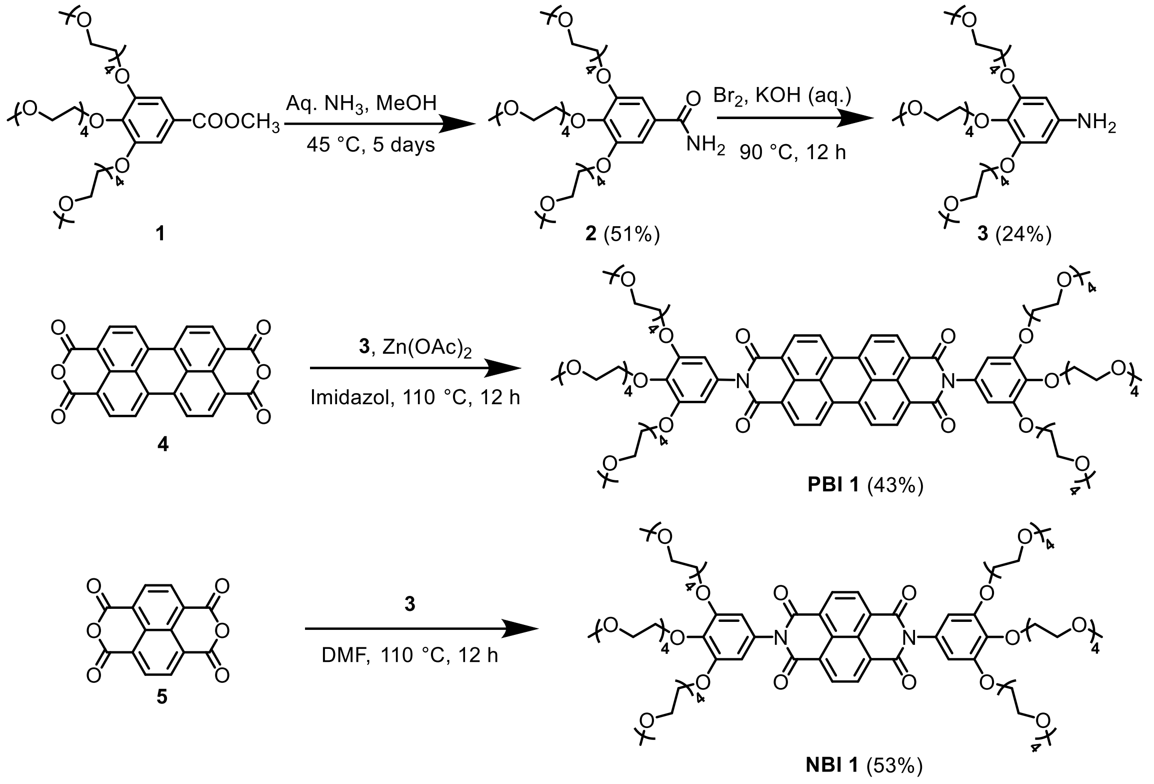

Scheme S1: Synthetic routes to **PBI 1** and **NBI 1**.

3,4,5-Tris((2,5,8,11-tetraoxatridecan-13-yl)oxy)benzamide (2)

Compound **1** (2 g, 2.65 mmol), aqueous NH_3 solution (25%,150 mL), and methanol (160 mL) were charged into a round-bottom flask fitted with a gas bubbler. The reaction mixture was stirred at 45 °C for 5 days. After the completion of the reaction, methanol was evaporated under reduced pressure and the product was extracted with dichloromethane (3x100 mL) and washed successively with water and brine solution. The organic phase was dried with anhydrous sodium sulphate. The residue obtained after evaporation of the solvent was purified by silica gel column chromatography (methanol 4%/chloroform) to obtain compound **2** as a pale yellow viscous oil (1.01 g, 1.37 mmol, 51%).

^{1}H NMR (400 MHz, $CDCl_3$): δ = 7.25 (s, 2H), 4.25-4.20 (m, 6H), 3.81-3.77 (m, 6H), 3.70-3.62 (m, 30H), 3.56-3.53 (m,6H), 3.38 (s, 6H), 3.37 (s, 3H) ppm. ^{13}C NMR (100 MHz, $CDCl_3$): δ = 168.8, 152.2, 142.0, 128.6, 108.6, 72.4, 71.98, 71.91, 70.7, 70.68, 70.64, 70.62, 70.57, 70.56, 70.54, 70.52, 69.8, 69.1, 59.09,

59.05 ppm. HRMS (ESI, positive, ACN/$CHCl_3$): *m*/*z* calcd for $C_{34}H_{61}NO_{16}Na$: 762.3888; found: 762.3888 $[M+Na]^+$.

3,4,5-Tris((2,5,8,11-tetraoxatridecan-13-yl)oxy)aniline (3)

Compound **2** (250 mg, 0.338 mmol) was added to an aqueous KOH solution (2M, 12 mL) in a round-bottom flask and the mixture was stirred for 15 minutes at 0 °C. Bromine in water solution (35 µL in 5 mL H_2O, 0.676 mmol) was added slowly to the suspension using a dropping funnel. After the addition was completed, the reaction mixture was stirred for 12 h at 90 °C and then cooled down to room temperature. The product was extracted with dichloromethane (3x50 mL) and washed with water and brine. The organic phase was dried with anhydrous sodium sulphate and the solvent was evaporated under vacuum. The residue was finally purified by silica gel column chromatography (methanol 4%/chloroform) to obtain the compound **3** as a yellow viscous oil (57 mg, 0.080 mmol, 24%).

^1H NMR (400 MHz, $CDCl_3$): δ = 5.95 (s, 2H), 4.07 (t, ^3J = 5.1 Hz, 4H), 4.00 (t, ^3J = 5.2 Hz, 2H), 3.78 (t, ^3J = 5.1 Hz, 4H), 3.72 (t, ^3J = 5.2 Hz, 2H), 3.70-3.67 (m, 6H), 3.65-3.60 (m, 24H), 3.53-3.50 (m, 6H), 3.35 (s, 9H) ppm. ^{13}C NMR (100 MHz, $CDCl_3$): δ = 153.2, 142.4, 131.2, 95.7, 72.4, 71.9, 70.8, 70.7, 70.64, 70.63, 70.61, 70.55, 70.52, 70.48, 69.7, 68.7, 59.0 ppm. HRMS (ESI, positive, MeOH): *m*/*z* calcd for $C_{33}H_{61}NO_{15}Na$: 734.3938; found: 734.3946 $[M+Na]^+$.

N,N'-[3,4,5-Tris((2,5,8,11-tetraoxatridecan-13-yl)oxy)phenyl] perylene-3,4:9,10-tetracarboxylic acid bisimide (PBI 1)

Perylene-3, 4: 9, 10-tetracarboxylic acid bisanhydride (**4**, 13.3 mg, 0.03 mmol), **3** (57 mg, 0. 08 mmol) and zinc acetate (25 mg, 0.13 mmol) were mixed with imidazole (1 g) and heated at 110 °C for 12 h under nitrogen. After cooling down to room temperature, the reaction mixture was dissolved in dichloromethane and washed first with 1M HCl followed by brine. The organic fraction was dried with anhydrous sodium sulphate and purified by column chromatography (methanol 4%/ chloroform), followed by preparative TLC (methanol 5%/ chloroform) and finally by GPC (chloroform) to obtain **PBI 1** as a red waxy solid. (26 mg, 0.01 mmol, 43%).

^1H NMR (400 MHz, $CDCl_3$): δ = 8.71 (d, 4H, ^3J = 7.9 Hz), 8.62 (d, 4H, ^3J = 8.1 Hz), 6.61 (s, 4H), 4.24 (t, ^3J = 5.1 Hz, 4H), 4.15 (t, ^3J = 4.9 Hz, 8H), 3.86-3.83 (m, 12H), 3.77–3.74 (m, 4H), 3.72–3.61 (m, 56H), 3.58–3.55 (m, 4H), 3.54–3.51 (m, 8H) 3.39 (s, 6H), 3.35 (s, 12H) ppm. ^{13}C NMR (100 MHz, $CDCl_3$): 163.0, 153.1, 138.3, 134.0, 131.2, 130.1, 128.8, 125.8, 123.2, 123.1, 108.0, 72.5, 72.05, 72.0, 70.8, 70.79, 70.73, 70.72, 70.70, 70.65, 70.63, 70.5, 69.6, 68.9, 59.17, 59.12 ppm. HRMS (ESI, positive, ACN/$CHCl_3$): *m*/*z* calcd for $C_{90}H_{128}N_2O_{34}^{2+}$: 890.4174; found: 890.4177 $[M+2H]^{2+}$. UV-vis ($CHCl_3$): λ_{max} = 528 nm (ε = 92200 M^{-1} cm^{-1}), 492 nm (ε = 55700 M^{-1} cm^{-1}).

N,*N'*-[3,4,5-Tris((2,5,8,11-tetraoxatridecan-13-yl)oxy)phenyl] naphthalene-1,4,5,8-tetracarboxylic acid bisimide (NBI 1)

1,4,5,8-Naphthalenetetracarboxylic acid bisanhydride (**5**, 45 mg, 0.16 mmol), 3,4,5-tris((2,5,8,11-tetraoxatridecan-13-yl)oxy) aniline (**3**, 300 mg, 0.42 mmol) and dry DMF (5 mL) were charged into a pressure tube and heated at 110 °C for 12 h under nitrogen. After cooling down to room temperature, the solvent was evaporated under vacuum and the residue was extracted with dichloromethane (3x50 mL) and washed with water and brine. The organic phase was dried with anhydrous sodium sulphate and then the solvent was evaporated under vacuum. The resultant residue was purified first by silica gel flash column chromatography (methanol 1.75%/chloroform) and then by GPC (chloroform) to obtain **NBI 1** as an orange waxy solid (149 mg, 0.09 mmol, 53%).

^{1}H NMR (400 MHz, $CDCl_3$): δ = 8.82 (s, 4H), 6.56 (s, 4H), 4.23 (t, ^{3}J = 5.1 Hz, 4H), 4.15 (t, ^{3}J = 5.0 Hz, 8H), 3.85-3.82 (m, 12H), 3.75-3.60 (m, 60H), 3.56-3.50 (m, 12H), 3.37 (s, 6H), 3.35 (s, 12H) ppm. ^{13}C NMR (100 MHz, $CDCl_3$): δ = 162.9, 153.3, 138.9, 131.5, 129.7, 127.2, 127.1, 108.2, 72.5, 72.05, 72.01, 70.9, 70.78, 70.75, 70.73, 70.70, 70.68, 70.66, 70.64, 70.60, 69.6, 69.0, 59.16, 59.14 ppm. HRMS (ESI, positive, ACN/$CHCl_3$): *m*/*z* calcd for $C_{80}H_{122}N_2O_{34}Na$: 1677.7776; found: 1677.7726 $[M+Na]^+$. UV-vis ($CHCl_3$): λ_{max} = 379 nm (ε = 26200 M^{-1} cm^{-1}), 359 nm (ε = 24000 M^{-1} cm^{-1}).

2. Synthesis of **PBI 2** and **NBI 2**

PBI 2 and **NBI 2** were prepared according to the synthetic route depicted in Scheme S2. The precursor 3,4,5-tris((2,5,8,11-tetraoxatridecan-13-yl)oxy)benzyl alcohol (**6**) was synthesized according to literature reported procedure.[8]

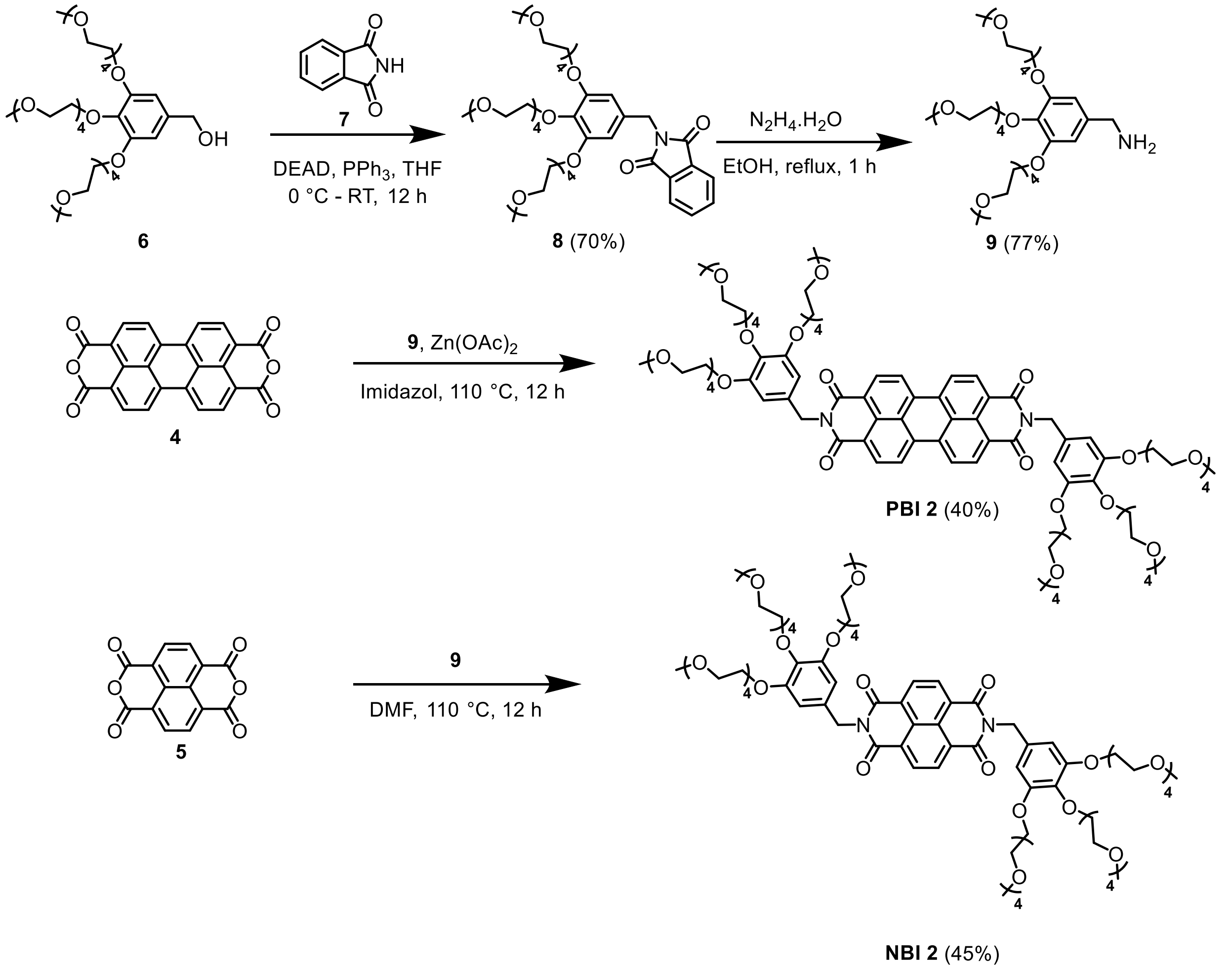

Scheme S2: Synthetic routes to **PBI 2** and **NBI 2**.

2-(3,4,5-Tris((2,5,8,11-tetraoxatridecan-13-yl)oxy)benzyl)isoindoline-1,3-dione (8)

Phthalimide (**7**, 456 mg, 3.1 mmol), triphenylphosphine (820 mg, 3.1 mmol) and **6** (1.47 g, 2.0 mmol) were suspended in 50 mL dry THF under nitrogen and cooled to 0 °C. To this suspension, a solution of DEAD (40% in toluene, 1.4 mL, 3.0 mmol) in 5 mL dry THF was added dropwise and was stirred overnight. The solvent was removed under vacuum and the resulting residue was purified by silica gel column chromatography (methanol 3%/chloroform) to obtain the product as a colorless oil (1.21 g, 1.41 mmol, 70%).

^{1}H NMR (400 MHz, $CDCl_3$): 7.81 (dd, ^3J = 5.5 Hz, ^4J = 2.9 Hz, 2H), 7.68 (dd, ^3J = 5.5 Hz, ^4J = 3.0 Hz, 2H), 6.65 (s, 2H), 4.69 (s, 2H), 4.10 (t, ^3J = 5.0 Hz, 4H), 4.05 (t, ^3J = 5.1 Hz, 2H), 3.79 (t, ^3J = 5.0 Hz, 4H) 3.72 (t, ^3J = 5.2 Hz, 2H) 3.69-3.59 (m, 30H), 3.52-3.49 (m, 6H), 3.34 (s, 6H), 3.33 (s, 3H) ppm.^{13}C NMR (100 MHz, $CDCl_3$): 168.0, 152.6, 138.0, 134.0, 132.1, 131.8, 123.4, 108.3, 72.2, 71.9, 70.8, 70.69, 70.64, 70.60, 70.56, 70.51, 69.7, 68.8, 59.0, 41.7 ppm. HRMS (MALDI, positive, $CHCl_3$): *m/z* calcd for $C_{42}H_{65}NO_{17}Na$: 878.4150; found: 878.4142 $[M+Na]^+$.

3,4,5-Tris((2,5,8,11-tetraoxatridecan-13-yl)oxy)benzyl amine (9)

To a solution of **8** (1.06 g, 1.24 mmol) in ethanol, 80 % hydrazine monohydrate (0.96 mL, 15.8 mmol) was added and refluxed for 1.5 h. The reaction mixture was filtered and evaporated under vacuum. The resultant residue was dissolved in dichloromethane, filtered off from the insoluble solids and washed with water followed by brine and was dried with anhydrous sodium sulphate. The solution was evaporated into dryness and the product was used for the next reaction without further purification (690 mg, 0. 95 mmol, 77%).

^{1}H NMR (400 MHz, $CDCl_3$): δ = 6.59 (s, 2H), 4.05 (t, ^3J = 4.8 Hz, 4H), 4.00 (t, ^3J = 4.9 Hz, 2H), 3.81 (s, 2H), 3.76 (t, ^3J = 4.8 Hz, 4H), 3.68 (t, ^3J = 4.9 Hz, 2H), 3.65-3.63 (m, 6H), 3.60-3.54 (m, 24H), 3.47-3.44 (m, 6H), 3.28 (s, 6H), 3.28 (s, 3H) ppm. ^{13}C NMR (100 MHz, $CDCl_3$): δ = 152.2, 136.7, 133.7, 107.2, 72.1, 71.82, 71.80, 71.7, 70.6, 70.54, 70.53, 70.47, 70.45, 70.42, 70.37, 70.34, 70.2, 69.6, 68.5, 58.9, 45.1 ppm. HRMS (ESI, positive, ACN/$CHCl_3$): *m/z* calcd for $C_{34}H_{63}NO_{15}Na$: 748.4089; found: 748.4081 $[M+Na]^+$.

N,N'-[3,4,5-Tris((2,5,8,11-tetraoxatridecan-13-yl)oxy)benzyl] perylene-3,4:9,10-tetracarboxylic acid bisimide (PBI 2)

Perylene-3, 4: 9, 10-tetracarboxylic acid bisanhydride (**4**, 64 mg, 0.16 mmol), **9** (300 mg, 0. 41 mmol) and zinc acetate (119 mg, 0.65 mmol) were mixed with imidazole (2 g) and heated at 110 °C for 12 h under nitrogen. After cooling down to room temperature, the reaction mixture was dissolved in dichloromethane and was extracted with 1M HCl and washed with brine. The organic fraction was purified by column chromatography (methanol 6%: chloroform) and by GPC (chloroform) to obtain **PBI 2** as a red waxy solid. (120 mg, 0.06 mmol, 40%).

^{1}H NMR (400 MHz, $CDCl_3$): 8.66 (d, 4H, ^3J = 8.0 Hz), 8.59 (d, 4H, ^3J = 8.1 Hz), 6.84 (s, 4H), 5.28 (s, 4H), 4.15 (t, ^3J = 5.1 Hz, 8H), 4.08 (t, ^3J = 5.2 Hz, 4H), 3.82 (t, ^3J = 5.0 Hz, 8H), 3.75–3.60 (m, 64H), 3.54–3.50 (m, 12H), 3.35 (s, 12H), 3.35 (s, 6H) ppm. ^{13}C NMR (100 MHz, $CDCl_3$): 163.4, 152.6, 138.0, 134.8, 132.5, 131.8, 129.5, 126.5, 123.3, 109.1, 72.3, 72.0, 70.9, 70.77, 70.70, 70.67, 70.63, 70.5, 69.8, 68.9, 59.1,

43.8 ppm. HRMS (ESI, positive, MeOH): *m/z* calcd for $C_{92}H_{130}N_2O_{34}Na_2^{2+}$: 926.4150; found: 926.4164 $[M+2Na]^{2+}$. UV-vis ($CHCl_3$): λ_{max} = 529 nm (ε = 88000 M^{-1} cm^{-1}), 492 nm (ε = 52000 M^{-1} cm^{-1}).

N,N'-[3,4,5-Tris((2,5,8,11-tetraoxatridecan-13-yl)oxy)benzyl] naphthalene-1,4,5,8-tetracarboxylic acid bisimide (NBI 2)

1,4,5,8-Naphthalenetetracarboxylic acid bisanhydride (**5**, 48 mg, 0.17 mmol), 3,4,5-tris((2,5,8,11-tetraoxatridecan-13-yl)oxy)benzyl amine (**9**, 356 mg, 0.49 mmol) and dry DMF (5 mL) were charged into a pressure tube and heated at 110 °C for 12 h under nitrogen. After cooling down to room temperature, the solvent was evaporated under vacuum and the residue was extracted with dichloromethane (3x50 mL) and washed with water and brine. The organic phase was dried with anhydrous sodium sulphate and then the solvent was evaporated under vacuum. The resultant residue was purified first by silica gel column chromatography (methanol 6%/chloroform) and then by GPC (chloroform) to obtain **NBI 2** as an orange waxy solid (136 mg, 0.08 mmol, 45%).

^{1}H NMR (400 MHz, $CDCl_3$): δ = 8.76 (s, 4H), 6.82 (s, 4H), 5.25 (s, 4H), 4.13 (t, ^3J = 5.1 Hz, 8H), 4.06 (t, ^3J = 5.1 Hz, 4H), 3.81 (t, ^3J = 5.0 Hz, 8H), 3.74-3.60 (m, 64H), 3.54-3.51 (m, 12H), 3.36 (s, 12H), 3.35 (s, 6H) ppm. ^{13}C NMR (100 MHz, $CDCl_3$): δ = 162.9, 152.6, 138.2, 132.0, 131.3, 126.8, 126.7, 109.3, 72.3, 72.0, 70.9, 70.76, 70.75, 70.71, 70.67, 70.64, 70.62, 70.5, 69.8, 68.9, 59.1, 44.1 ppm. HRMS (ESI, positive, ACN/$CHCl_3$): *m/z* calcd for $C_{82}H_{126}N_2O_{34}Na$: 1705.8089; found: 1705.8014 $[M+Na]^+$. UV-vis ($CHCl_3$): λ_{max} = 381 nm (ε = 26600 M^{-1} cm^{-1}), 361 nm (ε = 22500 M^{-1} cm^{-1}).

4. Monomer UV-vis spectra

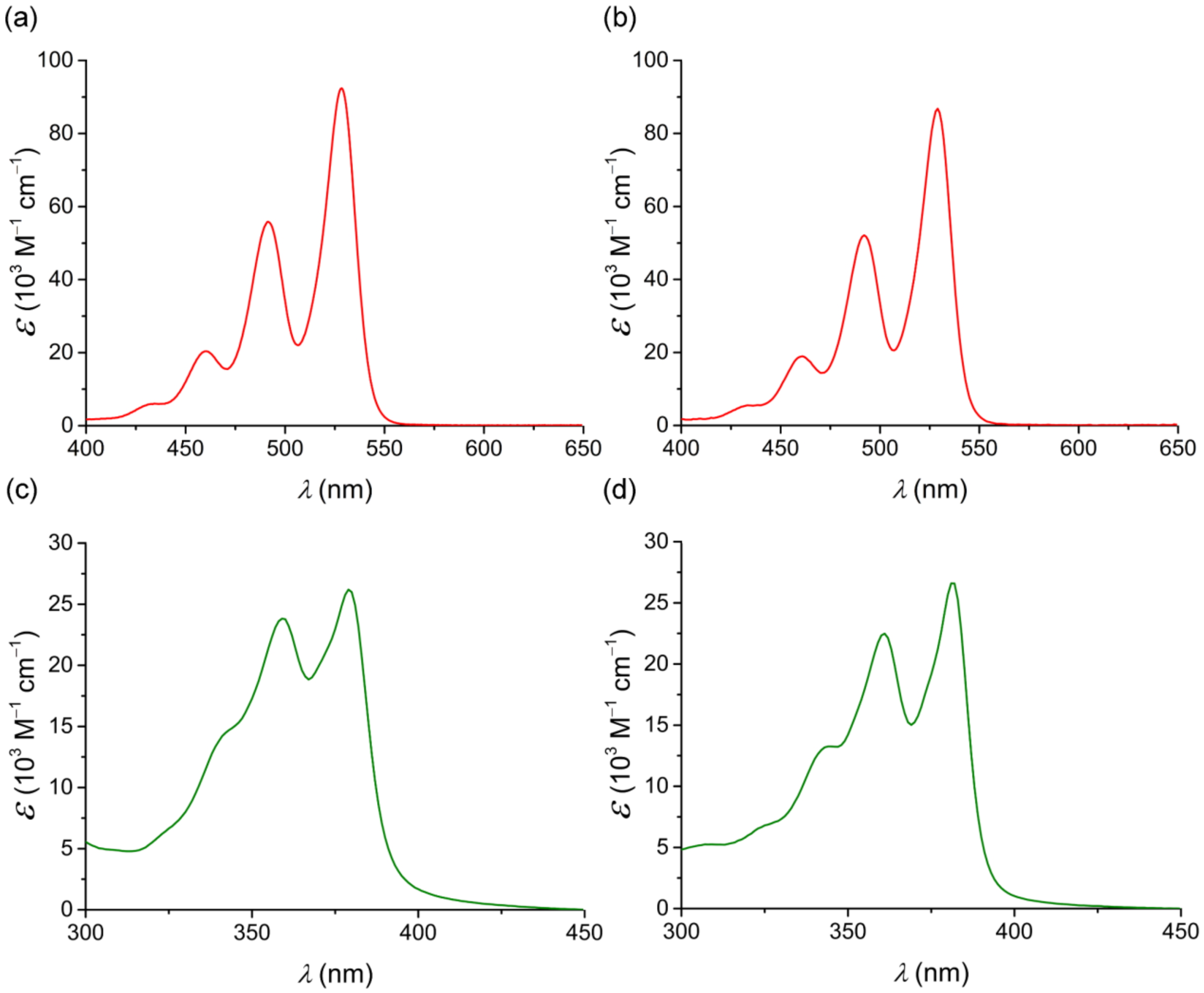

Figure S1. Monomer spectra of (a) **PBI 1** (c = 4.6 × 10^{-6} M), (b) **PBI 2** (c = 5.0 × 10^{-6} M), (c) **NBI 1** (c = 5.9 × 10^{-5} M) and (d) **NBI 2** (c = 5.7 × 10^{-5} M) in $CHCl_3$ at 25 °C.

5. Temperature-dependent UV-vis experiments

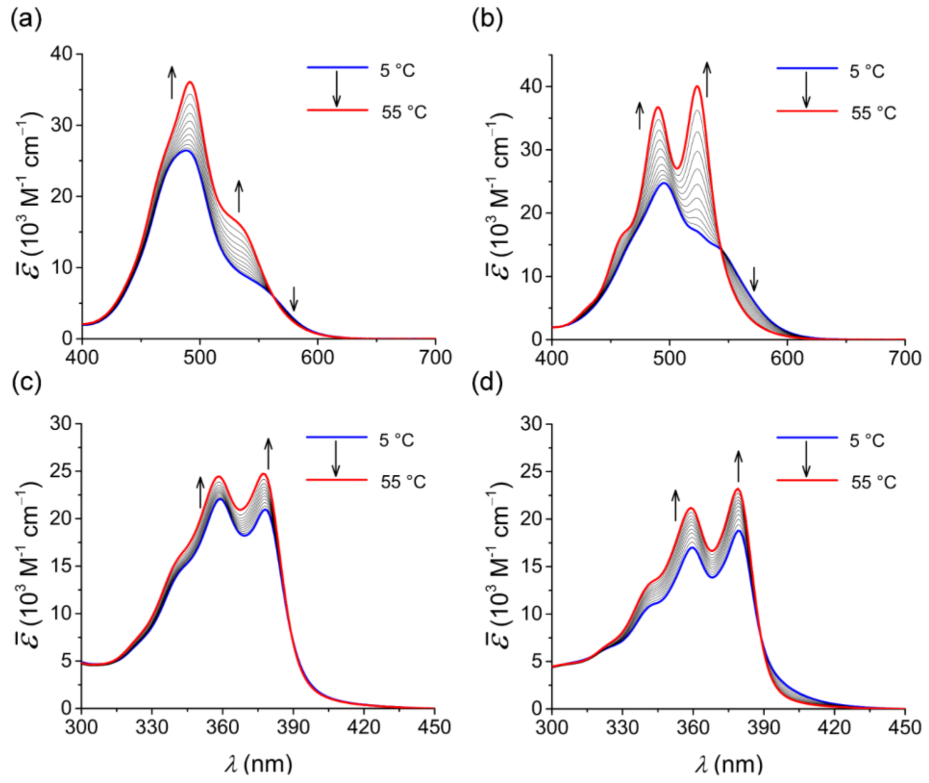

Figure S2. Temperature-dependent UV-vis spectra (density corrected) of (a) **PBI 1** ($c = 1.1 \times 10^{-4}$ M), (b) **PBI 2** ($c = 1.0 \times 10^{-4}$ M), (c) **NBI 1** ($c = 6.4 \times 10^{-4}$ M) and (d) **NBI 2 (**$c = 6.3 \times 10^{-4}$ M) in methanol. Arrows indicate spectral changes upon heating.

6. Atomic force microscopy (AFM)

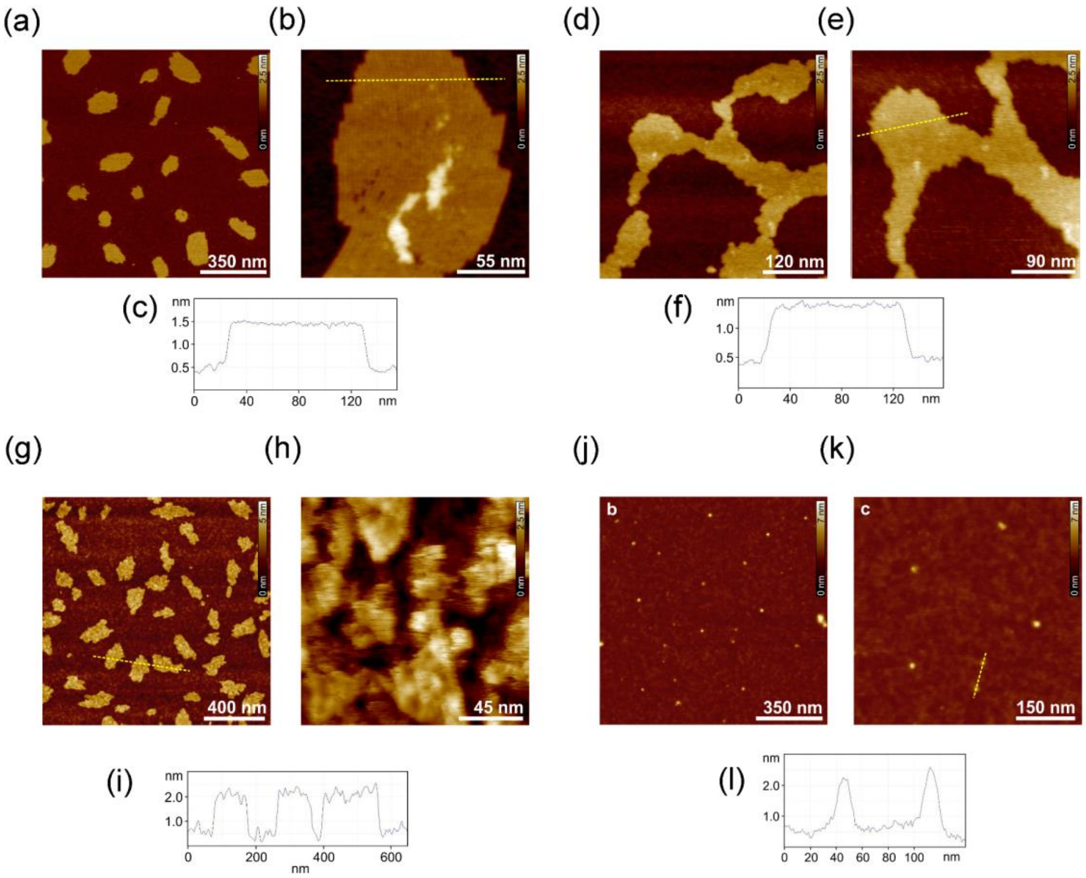

Figure S3. AFM height images of **PBI 1** ($c = 1.2 \times 10^{-4}$ M) (a, b), **PBI 2** ($c = 1.2 \times 10^{-4}$ M) (d, e) on mica and **NBI 1** ($c = 5.0 \times 10^{-3}$ M) (g, h), **NBI 2** ($c = 5.0 \times 10^{-3}$ M) (j, k) on silicon-wafer by spin-coating of aqueous solution at 22 °C. Corresponding cross-section analysis from the dashed yellow line in height images is also shown for **PBI 1** (c), **PBI 2** (f), **NBI 1** (i), and **NBI 2** (l).

7. Concentration-dependent UV-vis experiments

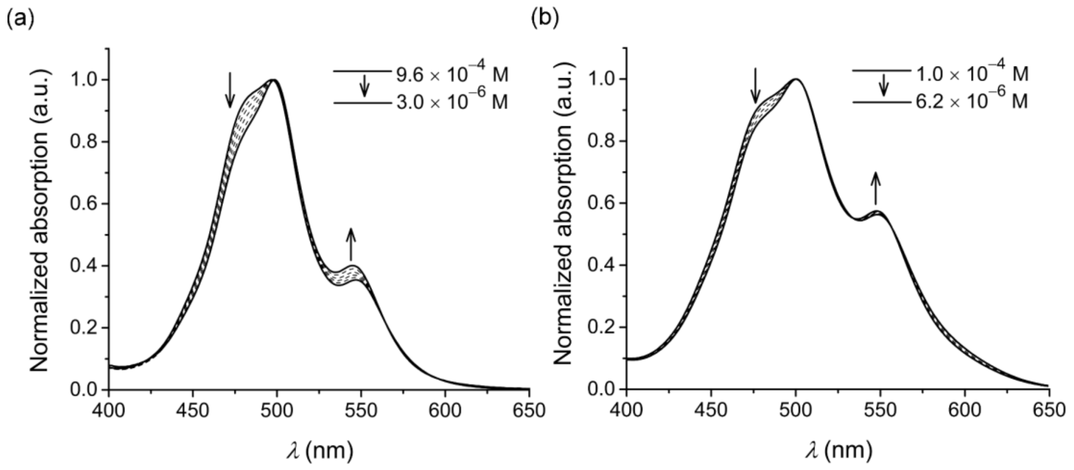

Figure S4. Concentration-dependent UV-vis spectra of (a) **PBI 1** and (b) **PBI 2** in water at 25 °C. Arrows indicate spectral changes upon dilution.

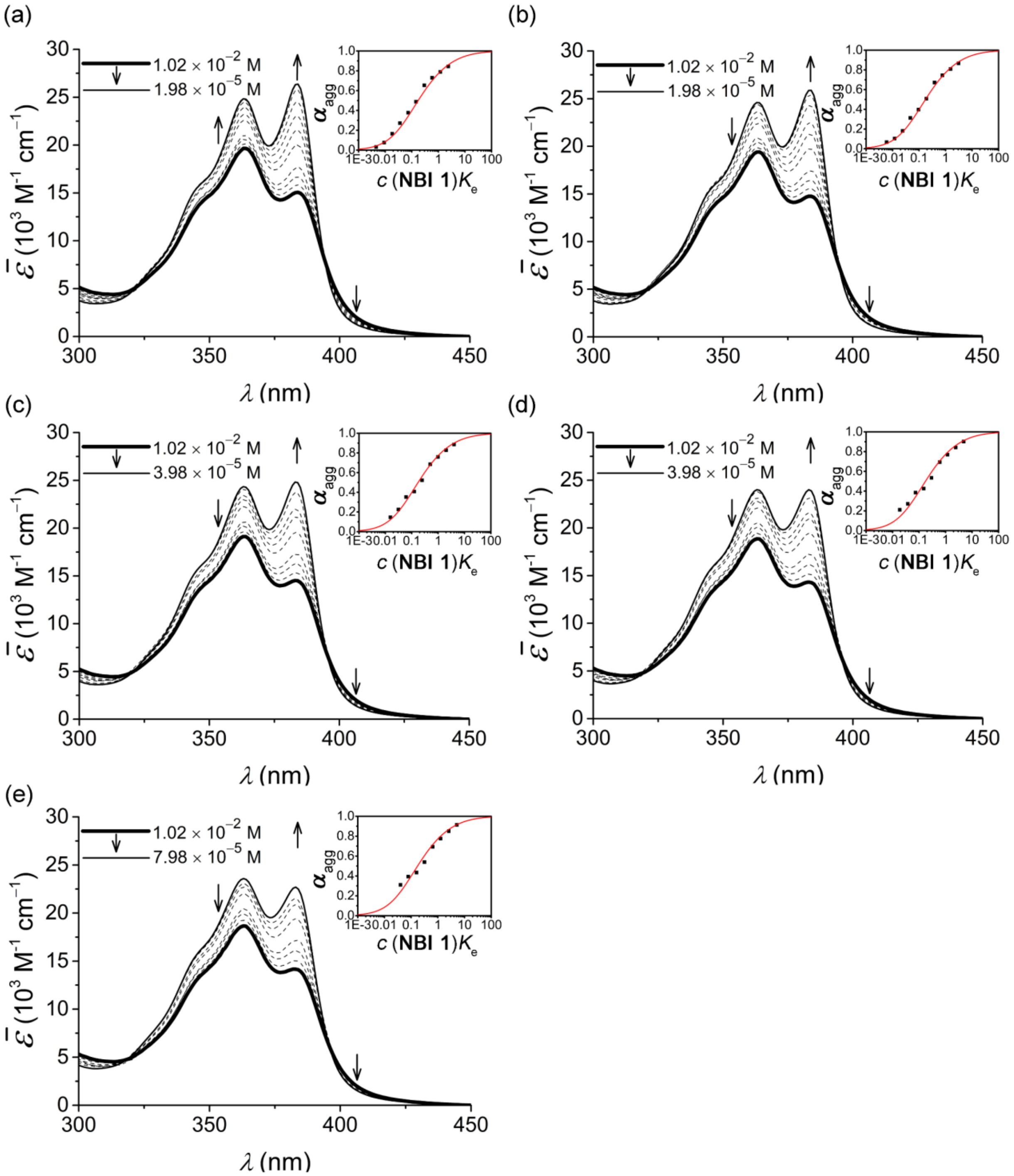

Figure S5. Concentration-dependent UV-vis spectra of **NBI 1** in water (density corrected) at (a) 10 °C, (b) 20 °C, (c) 30 °C, (d) 40 °C, and (e) 50 °C. Arrows indicate spectral changes upon dilution. Inset: Corresponding plot of fraction of aggregated species, α_{agg}, against dimensionless product c (**NBI 1**)K_e and analysis of the data based on the Goldstein-Stryer model (σ = 5).

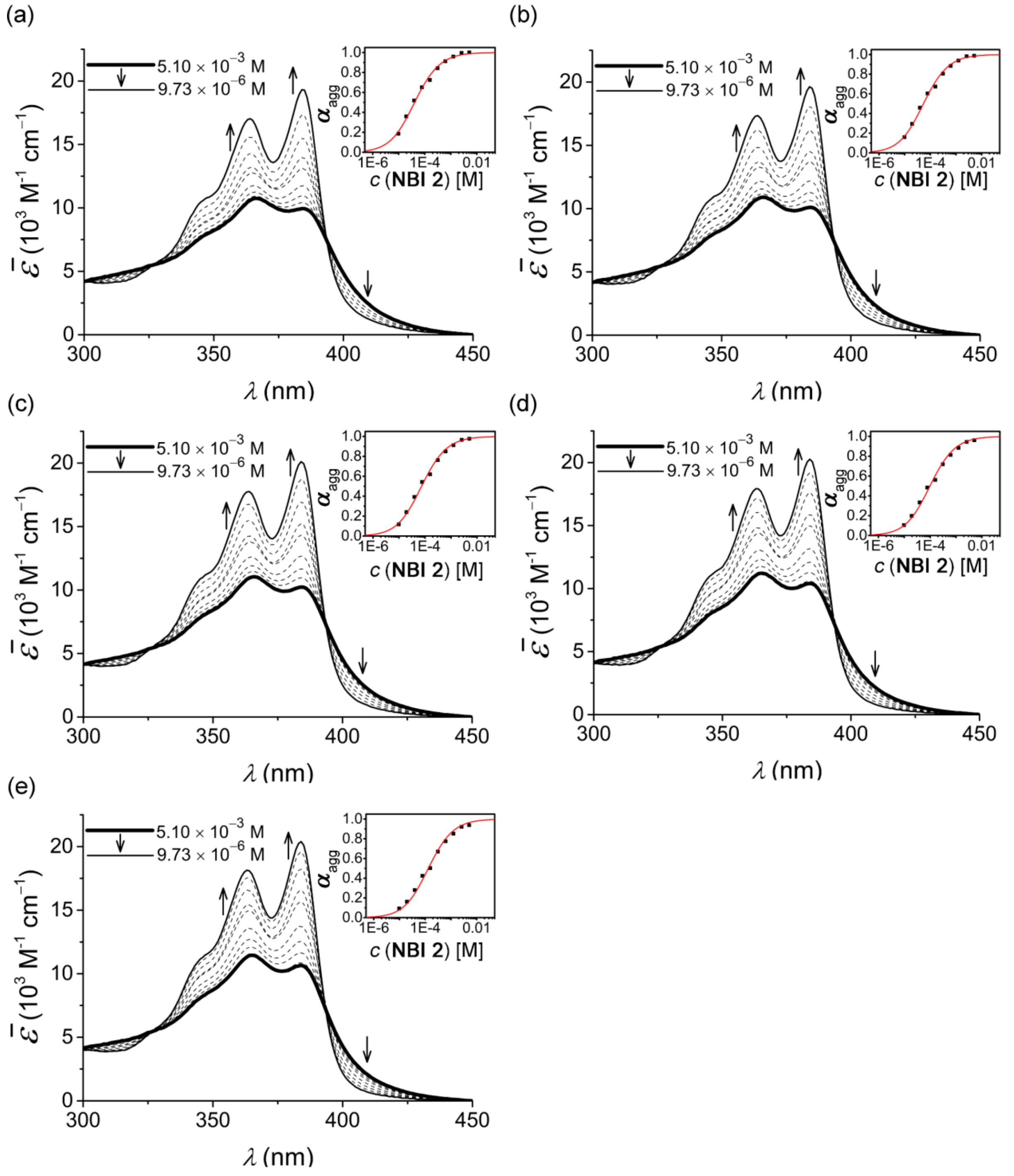

Figure S6. Concentration-dependent UV-vis spectra of **NBI 2** in water (density corrected) at (a) 10 °C, (b) 20 °C, (c) 30 °C, (d) 40 °C, and (e) 50 °C. Arrows indicate spectral changes upon dilution. Inset: Corresponding plot of fraction of aggregated species, α_{agg}, against concentration and analysis of the data based on the isodesmic model.

8. Isothermal titration calorimetry (ITC)

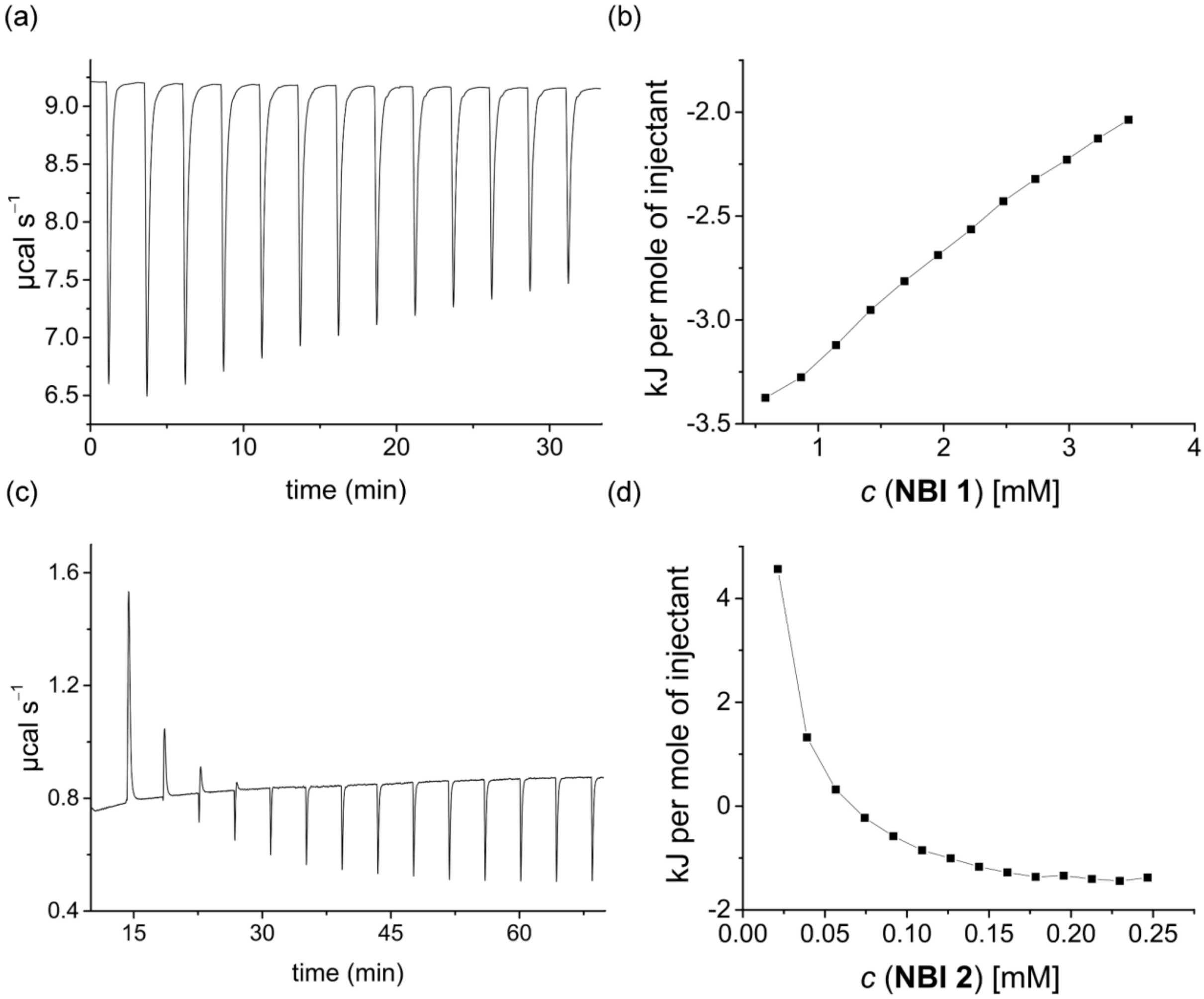

Figure S7. Evolution of heat per injection of (a) **NBI 1** ($c = 1.9 \times 10^{-2}$ M), (c) **NBI 2** ($c = 5.1 \times 10^{-3}$ M) in water at 25 °C and corresponding enthalpograms (b and d respectively). Lines connecting the data points are guide to eye.

9. NMR studies

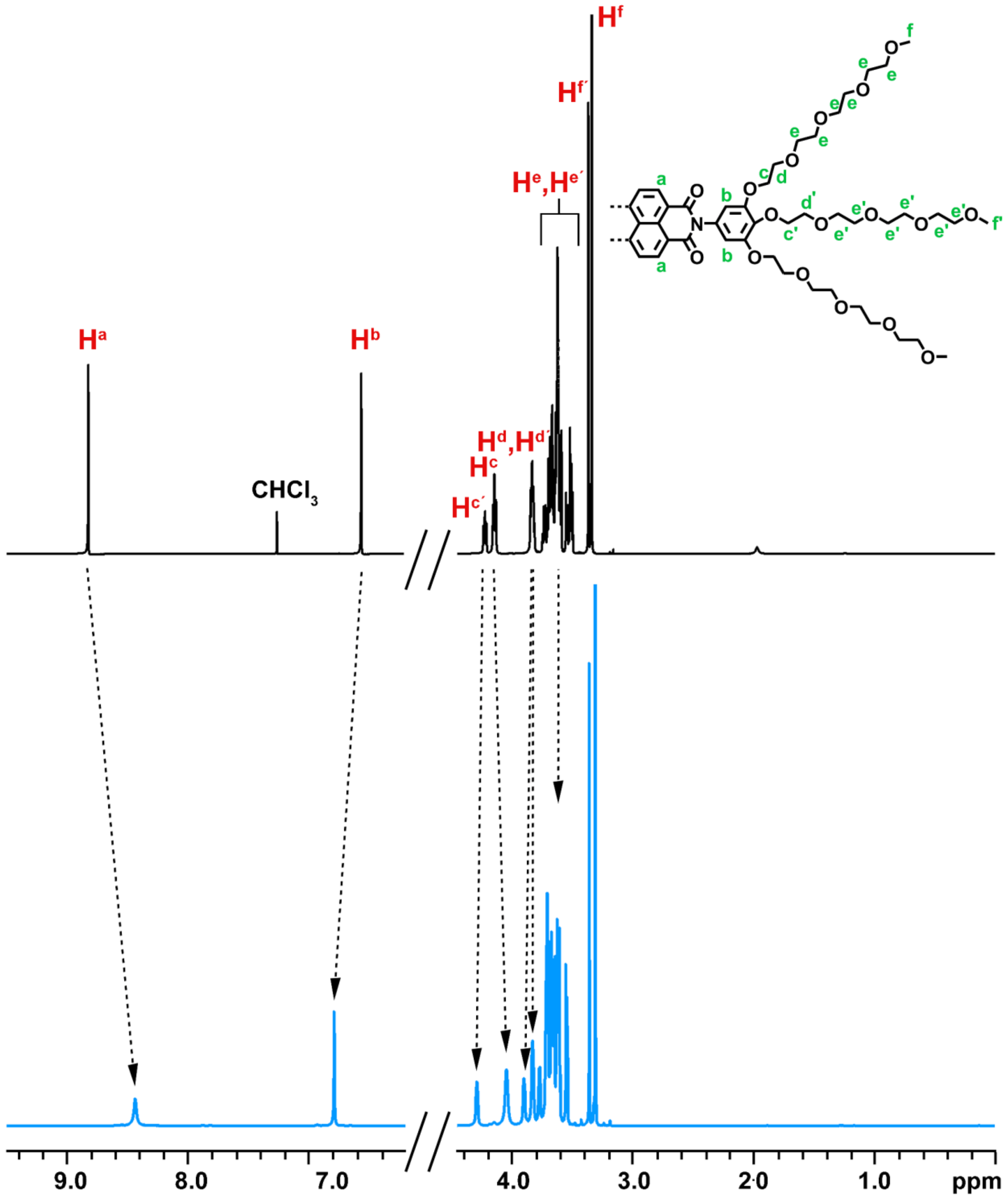

Figure S8. Relevant sections of ¹H NMR spectrum of **NBI 1** in $CDCl_3$ ($c = 11.0 \times 10^{-3}$ M) (top) and in D_2O ($c = 5.0 \times 10^{-3}$ M) (bottom) at 295 K. Partial chemical structure of **NBI 1** with the significant protons assigned is also shown.

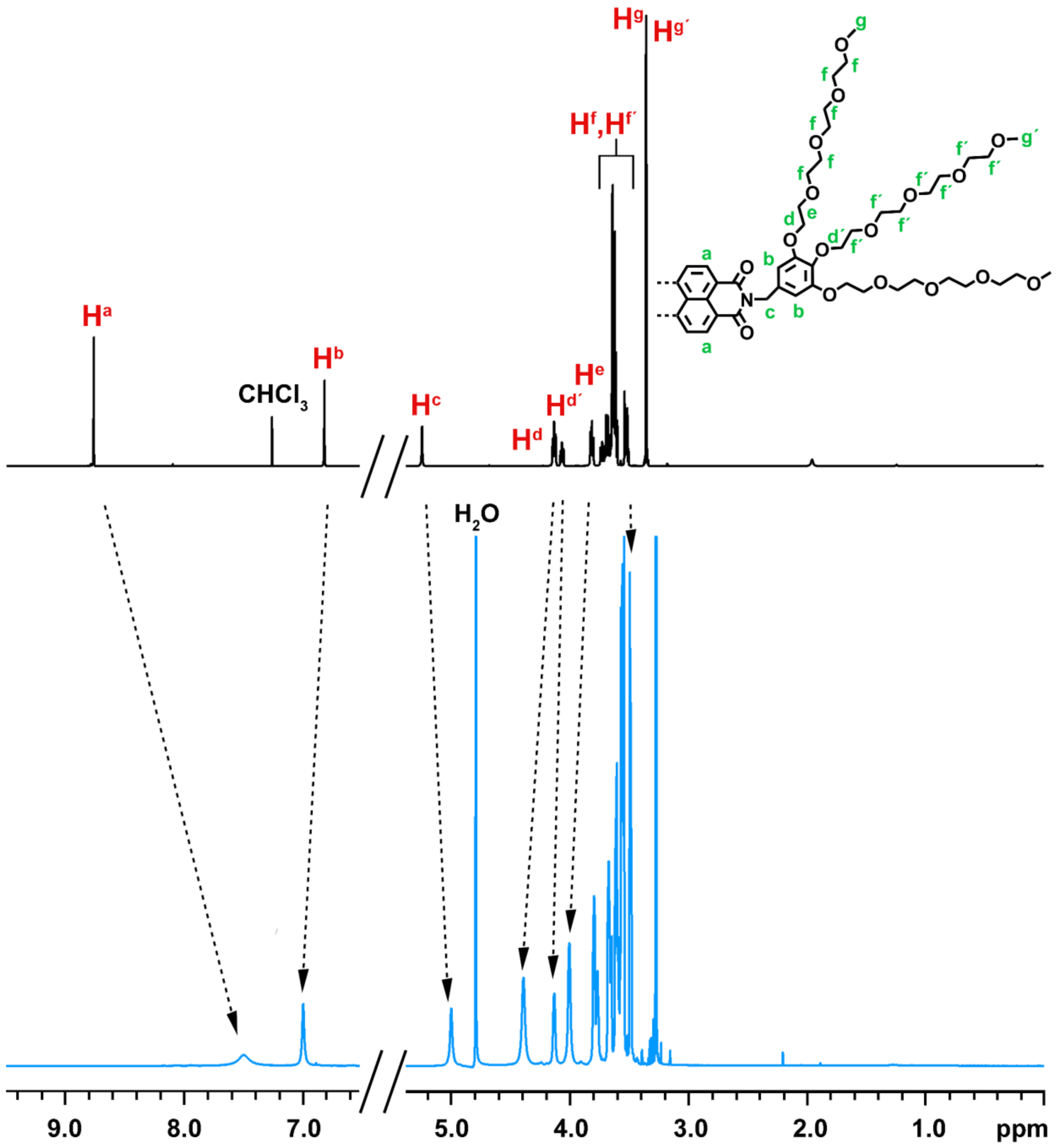

Figure S9. Relevant sections of ^{1}H NMR spectrum of **NBI 2** in $CDCl_3$ (c = 10.0 × 10^{-3} M) (top) and in D_2O (c = 4.9 × 10^{-3} M) (bottom) at 295 K. Partial chemical structure of **NBI 2** with the significant protons assigned is also shown.

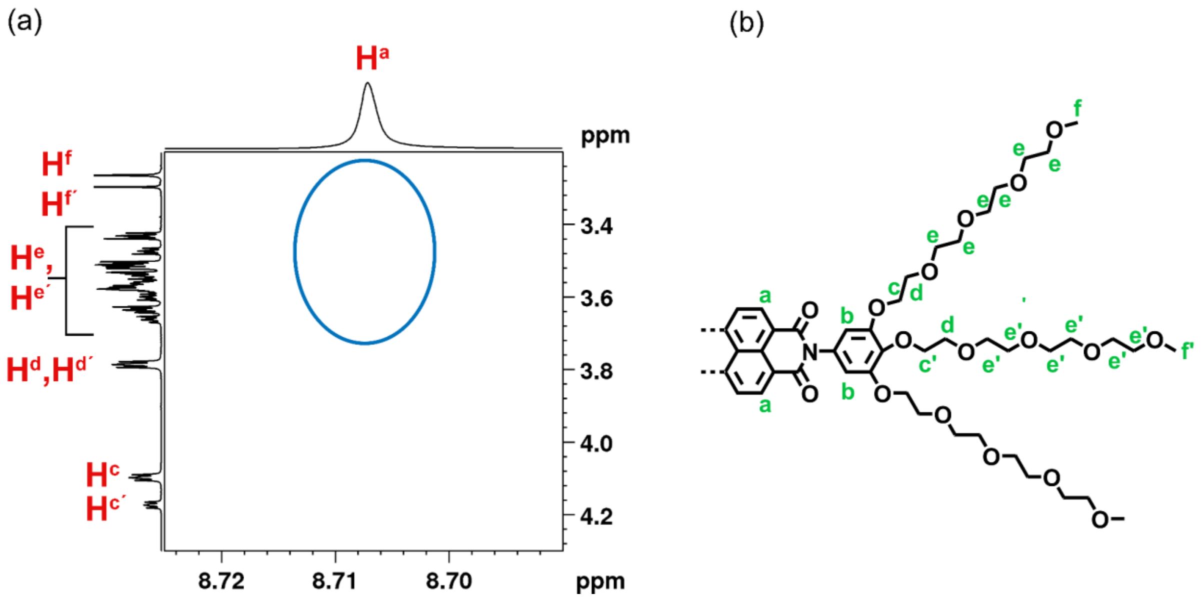

Figure S10. (a) Selected region of ^{1}H-^{1}H ROESY spectrum of **NBI 1** in CD_3CN (c = 6.9 × 10^{-3} M). Blue circle shows the region where NOEs corresponding to back-folding is to expect, but not observed. (b) Partial chemical structure of **NBI 1** with the significant protons assigned.

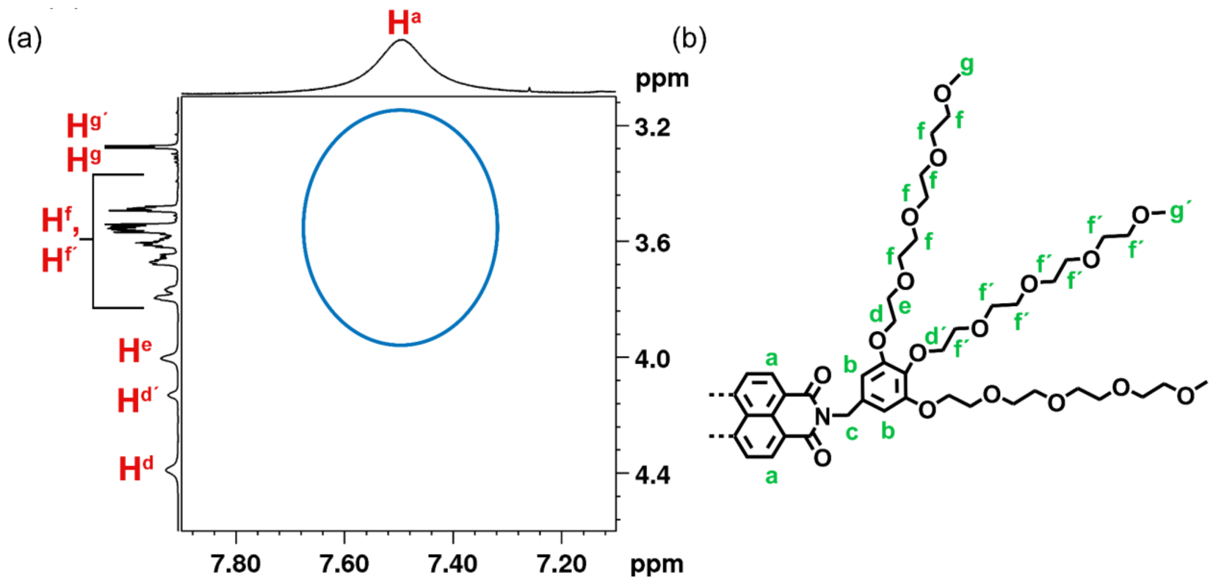

Figure S11. (a) Selected region of ^{1}H-^{1}H ROESY spectrum of **NBI 2** in D_2O (c = 4.9 × 10^{-3} M). Blue circle shows the region where NOEs corresponding to back-folding is to expect, but not observed. (b) Partial chemical structure of **NBI 2** with the significant protons assigned.

10.PM7 calculations

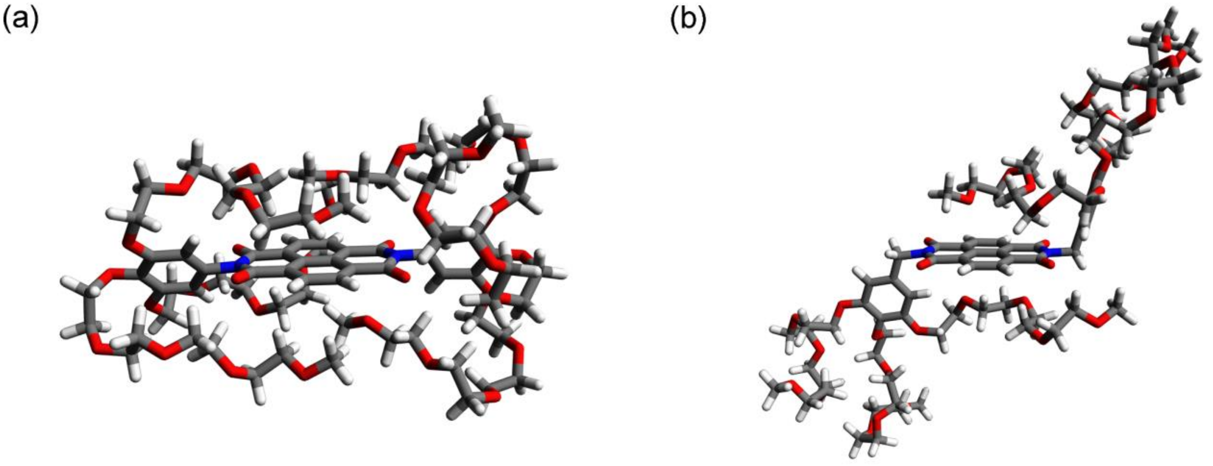

Figure S12. Geometry-optimized structures of (a) **NBI 1** and (b) **NBI 2** in water obtained by PM7 method in MOPAC.

11. NMR spectra of PBI 1, PBI 2 and NBI 1, NBI 2

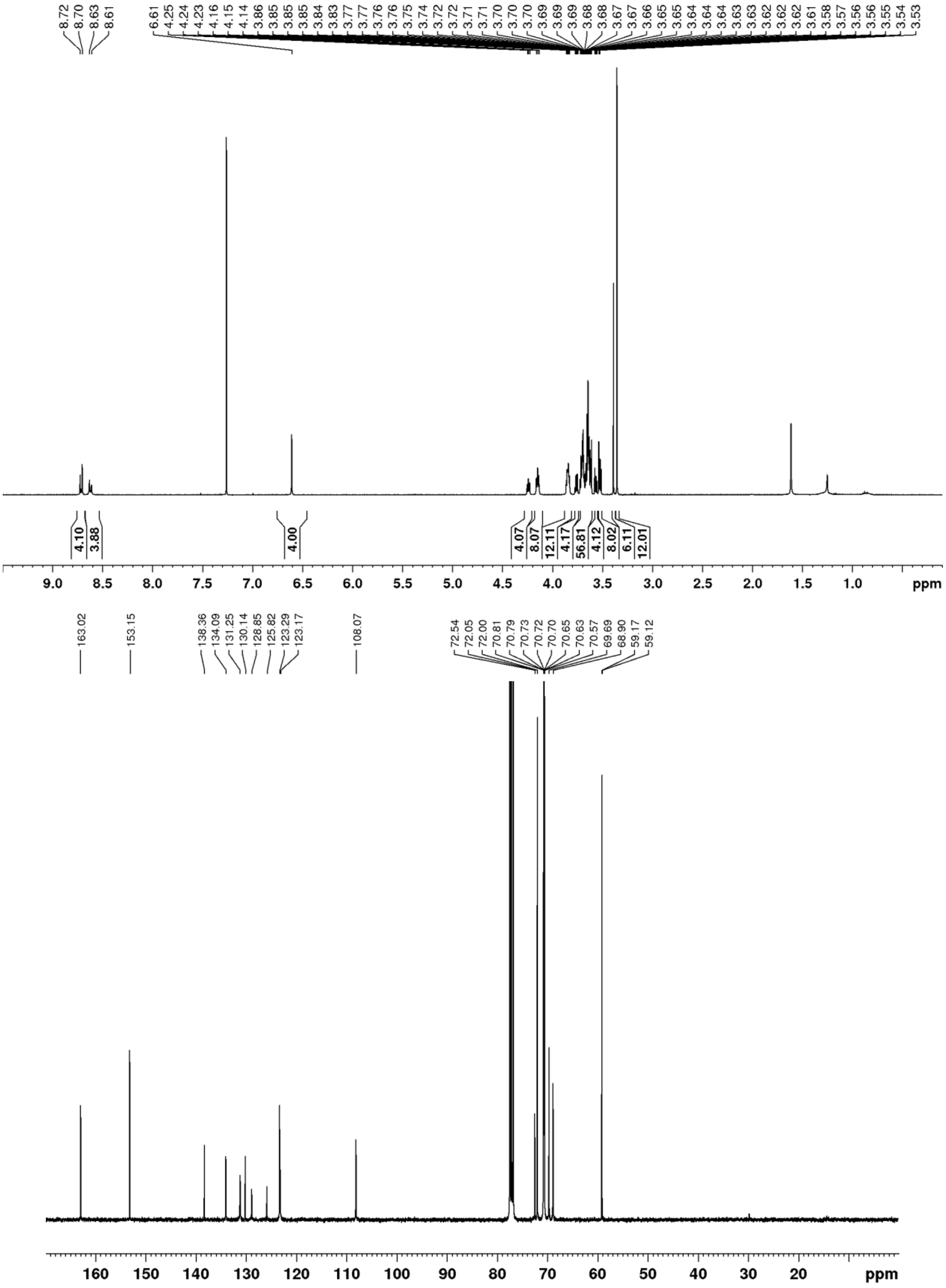

Figure S13. ^{1}H (400 MHz, top) and ^{13}C NMR spectra (100 MHz, bottom) of **PBI 1** in $CDCl_3$ at 295 K.

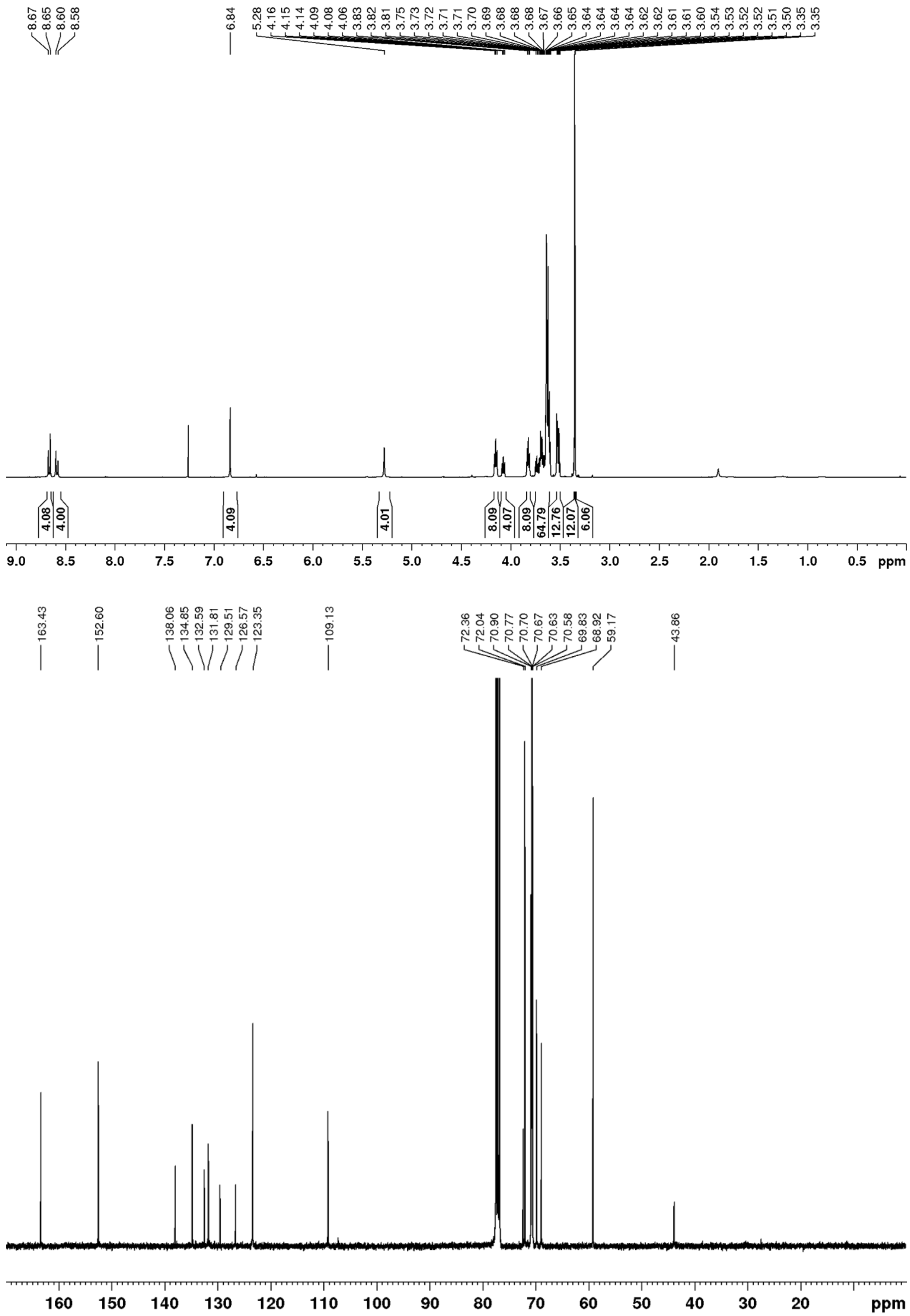

Figure S14. ^{1}H (400 MHz, top) and ^{13}C NMR spectra (100 MHz, bottom) of **PBI 2** in $CDCl_3$ at 295 K.

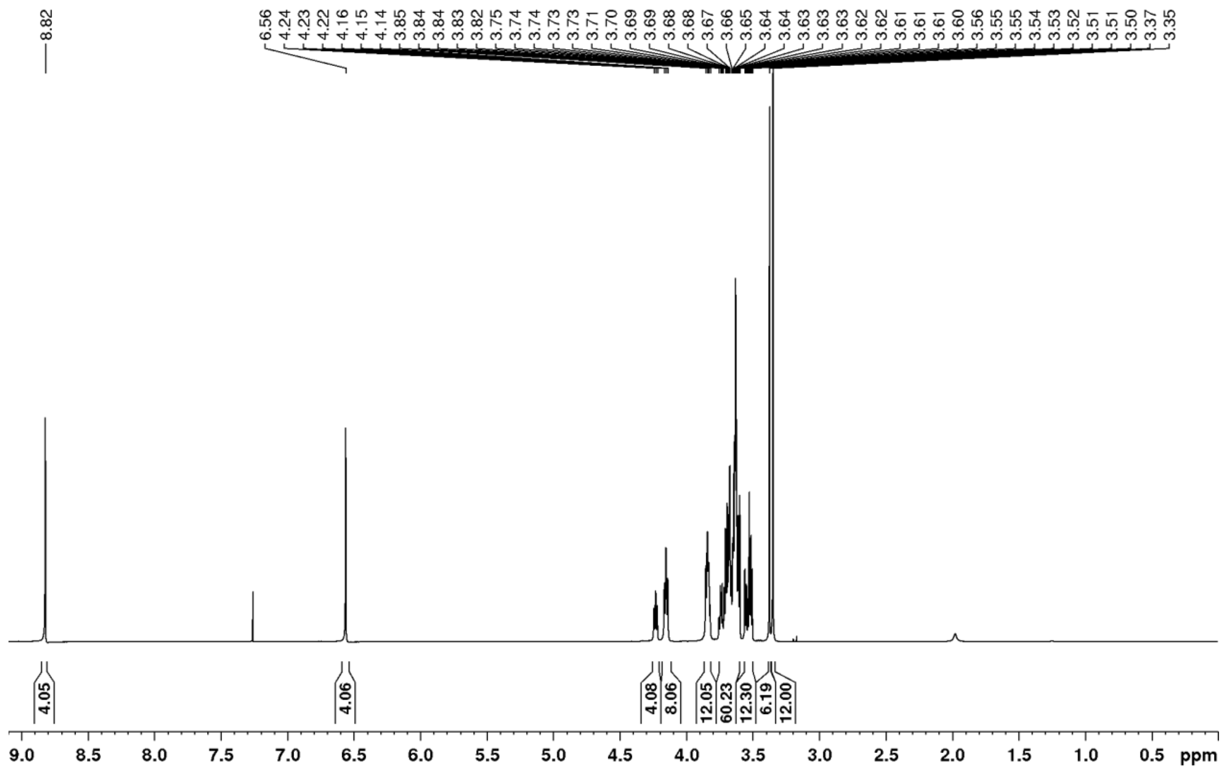

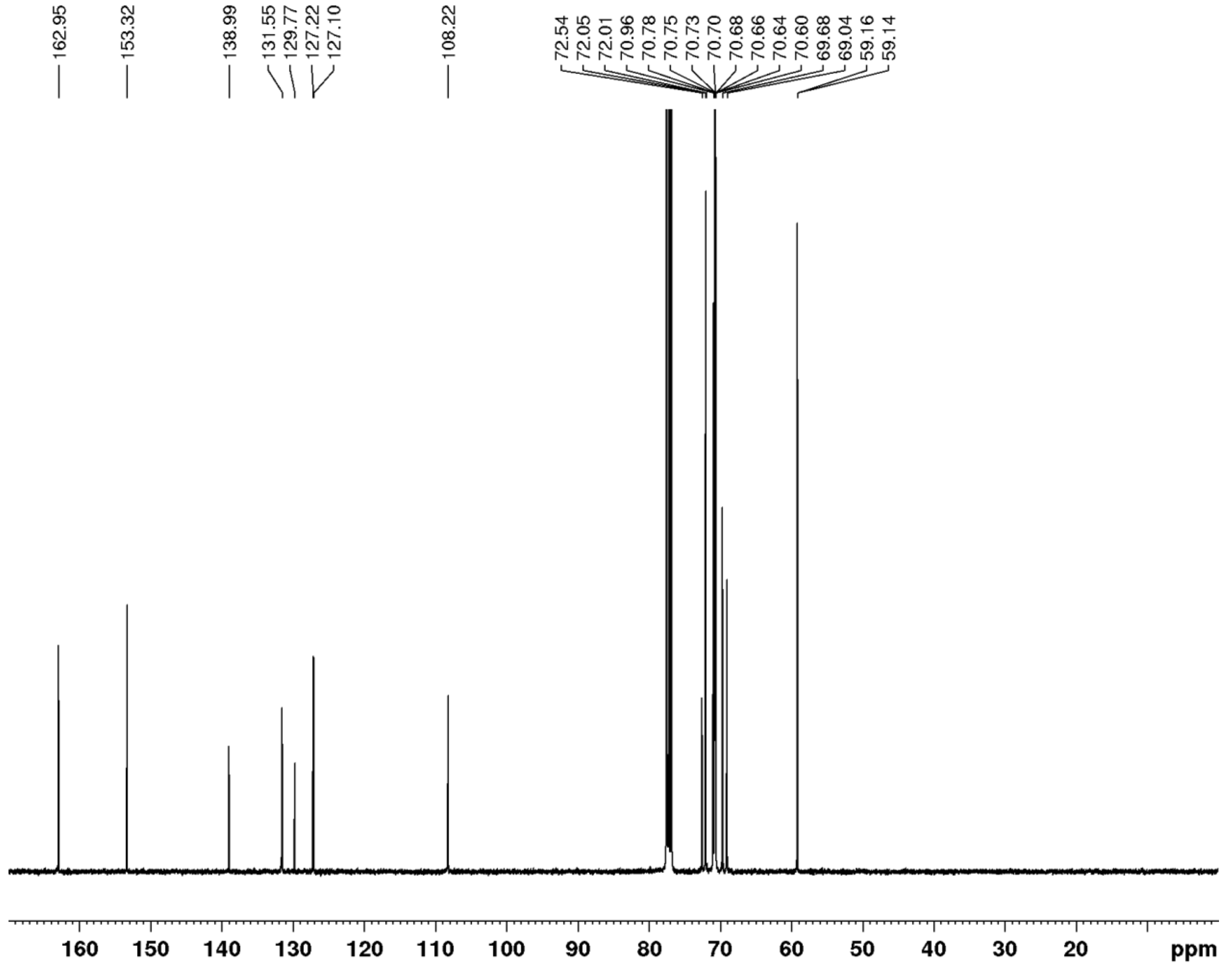

Figure S15. ^{1}H (400 MHz, top) and ^{13}C NMR spectra (100 MHz, bottom) of **NBI 1** in $CDCl_3$ at 295 K.

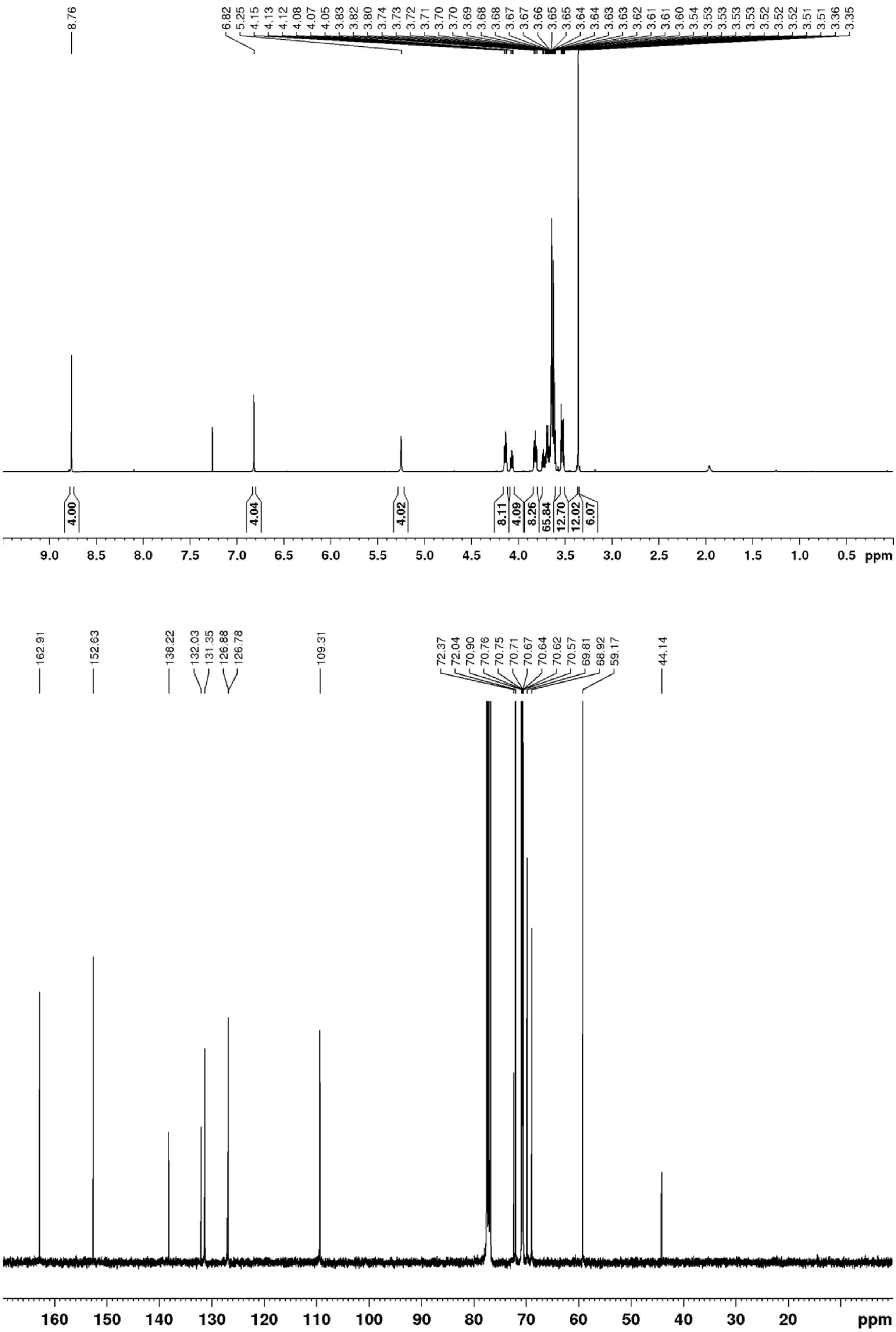

Figure S16. ^{1}H (400 MHz, top) and ^{13}C NMR spectra (100 MHz, bottom) of **NBI 2** in $CDCl_3$ at 295 K.

12. References

[1] J. J. P. Stewart, *J. Mol. Model.* **2013**, *19*, 1-32.

[2] J. J. P. Stewart, *Stewart Computational Chemistry* **2016**, *Colorado Springs, CO, USA. HTTP://OpenMOPAC.net*.

[3] A. Klamt, G. Schüürmann, *J. Chem. Soc., Perkin Trans. 2* **1993**, 799-805.

[4] Z. Chen, A. Lohr, C. R. Saha-Möller, F. Würthner, *Chem. Soc. Rev.* **2009**, *38*, 564-584.

[5] R. F. Goldstein, L. Stryer, *Biophys. J.* **1986**, *50*, 583-599.

[6] M. J. Mayoral, C. Rest, V. Stepanenko, J. Schellheimer, R. Q. Albuquerque and G. Fernández, *J. Am. Chem. Soc.*, **2013**, *135*, 2148-2151.

[7] P. Rajdev, M. R. Molla, S. Ghosh, *Langmuir* **2014**, *30*, 1969-1976.

[8] M. D. Tzirakis, M. N. Alberti, H. Weissman, B. Rybtchinski, F. Diederich, *Chem. Eur. J.* **2014**, *20*, 16070-16073.
